# Some Phthalocyanine and Naphthalocyanine Derivatives as Corrosion Inhibitors for Aluminium in Acidic Medium: Experimental, Quantum Chemical Calculations, QSAR Studies and Synergistic Effect of Iodide Ions

**DOI:** 10.3390/molecules200915701

**Published:** 2015-08-28

**Authors:** Masego Dibetsoe, Lukman O. Olasunkanmi, Omolola E. Fayemi, Sasikumar Yesudass, Baskar Ramaganthan, Indra Bahadur, Abolanle S. Adekunle, Mwadham M. Kabanda, Eno E. Ebenso

**Affiliations:** 1 Department of Chemistry, School of Mathematical & Physical Sciences, North-West University (Mafikeng Campus), Private Bag X2046, Mmabatho 2735, South Africa; E-Mails: masegodibetsoe@yahoo.com (M.D.); waleolasunkanmi@gmail.com (L.O.O.); fomololaesther@yahoo.com (O.E.F.); sasikumar.phd@gmail.com (S.Y.); ramaganthanbaskar@gmail.com (B.R.); bahadur.indra@gmail.com (I.B.); sadekpreto@gmail.com (A.S.A.); Mwadham.Kabanda@nwu.ac.za (M.M.K.); 2 Material Science Innovation & Modelling (MaSIM) Research Focus Area, Faculty of Agriculture, Science and Technology, North-West University (Mafikeng Campus), Private Bag X2046, Mmabatho 2735, South Africa; 3 Department of Chemistry, Faculty of Science, Obafemi Awolowo University, Ile-Ife 220005, Nigeria

**Keywords:** phthalocyanines, naphthalocyanines, corrosion inhibitors, aluminium, quantum chemical calculations, QSAR, peripheral groups

## Abstract

The effects of seven macrocyclic compounds comprising four phthalocyanines (Pcs) namely 1,4,8,11,15,18,22,25-octabutoxy-29*H*,31*H*-phthalocyanine (Pc1), 2,3,9,10,16,17,23,24-octakis(octyloxy)-29*H*,31*H*-phthalocyanine (Pc2), 2,9,16,23-tetra-*tert*-butyl-29*H*,31*H*-phthalocyanine (Pc3) and 29*H*,31*H*-phthalocyanine (Pc4), and three naphthalocyanines namely 5,9,14,18,23,27,32,36-octabutoxy-2,3-naphthalocyanine (nPc1), 2,11,20,29-tetra-*tert*-butyl-2,3-naphthalocyanine (nPc2) and 2,3-naphthalocyanine (nP3) were investigated on the corrosion of aluminium (Al) in 1 M HCl using a gravimetric method, potentiodynamic polarization technique, quantum chemical calculations and quantitative structure activity relationship (QSAR). Synergistic effects of KI on the corrosion inhibition properties of the compounds were also investigated. All the studied compounds showed appreciable inhibition efficiencies, which decrease with increasing temperature from 30 °C to 70 °C. At each concentration of the inhibitor, addition of 0.1% KI increased the inhibition efficiency compared to the absence of KI indicating the occurrence of synergistic interactions between the studied molecules and I^−^ ions. From the potentiodynamic polarization studies, the studied Pcs and nPcs are mixed type corrosion inhibitors both without and with addition of KI. The adsorption of the studied molecules on Al surface obeys the Langmuir adsorption isotherm, while the thermodynamic and kinetic parameters revealed that the adsorption of the studied compounds on Al surface is spontaneous and involves competitive physisorption and chemisorption mechanisms. The experimental results revealed the aggregated interactions between the inhibitor molecules and the results further indicated that the peripheral groups on the compounds affect these interactions. The calculated quantum chemical parameters and the QSAR results revealed the possibility of strong interactions between the studied inhibitors and metal surface. QSAR analysis on the quantum chemical parameters obtained with B3LYP/6-31G (d,p) method show that a combination of two quantum chemical parameters to form a composite index provides the best correlation with the experimental data.

## 1. Introduction

Aluminium (Al) and its alloys are often considered as preferred materials for applications in various industries, especially those that deal with automobiles, household appliances, aluminium containers, electronic devices, building, aviation, *etc.* This is because of the unique properties of Al, which include low density, lustrous appearance, relatively good corrosion resistance as well as excellent thermal and electrical conductivity [1,2,3]. Al has the intrinsic characteristic of exhibiting initial corrosion resistance in aqueous environments due to the formation of passive oxide film on its surface. The passivating natural oxide film comprises essentially aluminium oxides with amphoteric characteristics. As a result, the oxide film dissolves in acidic or basic environments and the metal once again becomes exposed to corrosive attack in these environments. The dissolution of Al has been reported to be more rapid in acidic solutions containing chloride or fluoride ions than in the near-neutral solutions [4,5,6]. Corrosion inhibitors offer an efficient, a convenient and relatively cheap method of controlling corrosion of metals in various aggressive media [3,7,8,9,10,11]. For this reason, a number of studies have been reported on the use of corrosion inhibitors to mitigate the corrosion of Al in chloride solutions [12,13,14].

Organic molecules with π-electron systems and highly electronegative atoms such as O, N, S and P are generally considered as prospective corrosion inhibitors. The inhibition of metal corrosion by these compounds is usually premised on their ability to adsorb on metallic surface. Various factors such as the electronic structure of the organic compound, planarity of the molecule, steric hindrance, aromaticity *etc.* are known to affect the inhibition potential of organic corrosion inhibitors. The phthalocyanine is a planar organic molecule with eight N atoms in a macrocyclic nucleus comprising an extended conjugated π-electron systems and aromatic rings. Phthalocyanines possess intense optical absorption in the red/near infra-red region of solar spectrum making them to exhibit excellent light-harvesting characteristics and suitable for the design of photovoltaic devices [15,16]. Their distinct physicochemical properties and the unique redox chemistry of their metal complexes have brought about their incorporation into a large number of donor-acceptor systems in which they function efficiently as light-harvesting substances and electron donors to an acceptor unit [17]. Their metal complexes have also been reportedly used for various photocatalytic and electrocatalytic applications [18,19,20].

Phthalocyanines and other porphyrinoid compounds have been successfully used as corrosion inhibitors in a number of studies [21,22,23,24]. Their efficient corrosion inhibition potentials are partly due to the planarity of their molecules and their large molecular volume which enhances their adsorption onto metal surface and informed a large degree of surface coverage. In addition to their efficient corrosion inhibition properties, phthalocyanines are cost effective, easy to synthesize, thermally stable and chemically inert [25,26]. Solubility and aggregation are another important properties of phthalocyanines which in turn can influence there adsorption and corrosion inhibition properties. The solubility of phthalocyanines in various solvents largely depends on the substituents groups or coordinated ligands and can be tuned by peripheral design. On the other hand, aggregation is a function of phthalocyanine chromophore and results mainly from the attractive interaction between two or more molecules [27].

The limited solubility of macrocylic compounds and polymers in aqueous environment also limits their corrosion inhibition potentials. This is because inhibitors that are completely soluble in the medium of application generally exhibit higher efficiency than the less soluble ones. Therefore, these molecules are often utilized at relatively low concentrations and one common method of improving their inhibition efficiency at low concentrations is the use of auxiliary compounds or species that can synergistically improve their performance as corrosion inhibitors. In this regard, halides are often used as synergistic materials with macrocylic and polymeric corrosion inhibitors. Previous reports on synergism in corrosion inhibition studies have shown that iodide (I^−^) ions give the best results among the halides [28,29,30]. Hence, researchers usually focus on the synergistic inhibitive effect of I^−^ ions [31,32].

**Figure 1:**
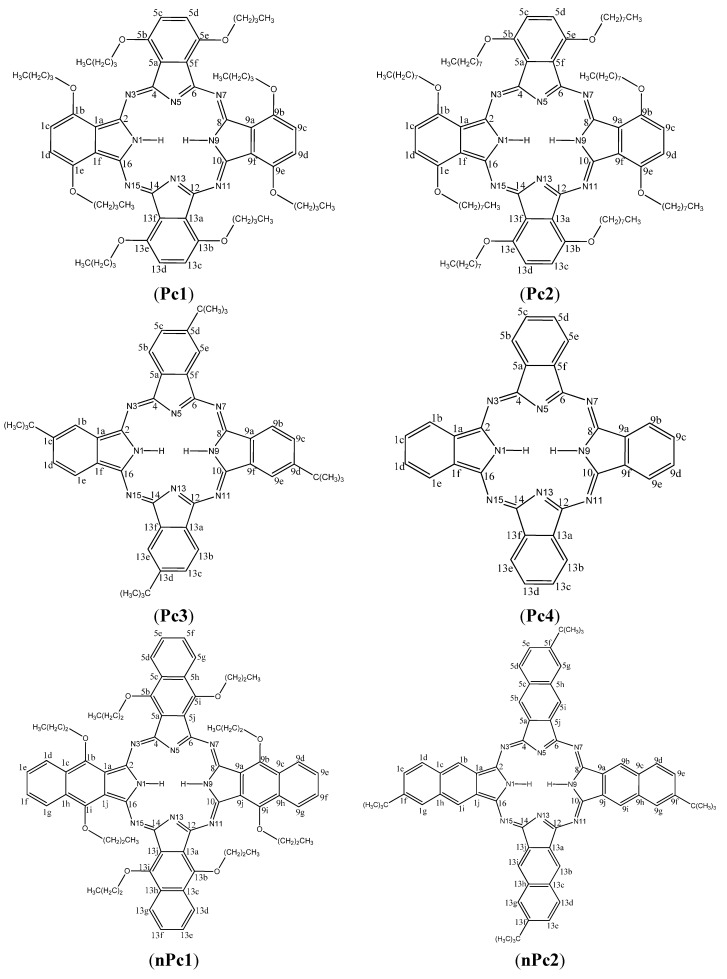

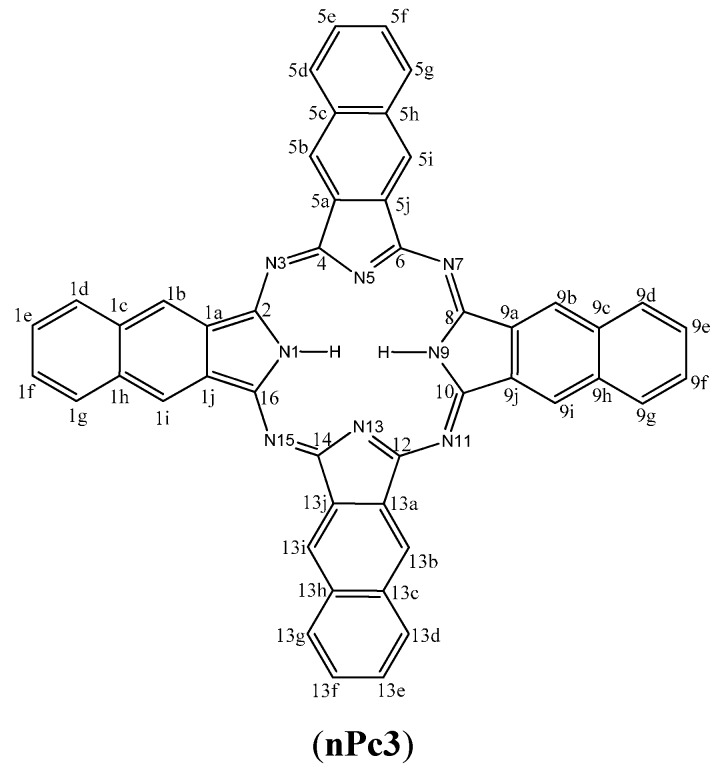
Molecular structures of the studied phthalocyanines (Pcs) and naphthalocyanines (nPcs): (**Pc1**): 1,4,8,11,15,18,22,25-octabutoxy-29*H*,31*H-*phthalocyanine; (**Pc2**): 2,3,9,10,16,17,23,24-octakis(octaloxy)-29*H*,31*H*-phthalocyanine; (**Pc3**): 2,9,16,23-tetra-*tert*-butyl-29*H*,31*H*-phthalocyanine; (**Pc4**): naphthalocyanine (**nPc2**)29*H*,31*H*-Phthalocyanine; (**nPc1**): 5,9,14,18,23,27,32,36-octabutoxy-2,3-naphthalocyanine; (**nPc2**): 2,11,20,29-tetra-*tert*-butyl-2,3-(Pc4); (**nPc3**): 2,3-naphthalocyanine.

Reports on comparative corrosion inhibition potentials of series of phthalocyanines and/or naphthalocyanines comprising experimental, quantum chemical and quantitative structure activity relationship (QSAR) studies are not common. More so, studies that attempt to explore the complex aggregative interactions of macrocylic compounds in aqueous acidic environments in combination with synergistic interactions with KI and the resulting effects on corrosion inhibition potentials are very scanty. This has motivated us to carry out the present study to investigate the inhibition potentials of seven macrocyclic compounds comprising four phthalocyanines and three naphthalocyanines namely, 1,4,8,11,15,18,22,25-octabutoxy-29*H*,31*H*-phthalocyanine (Pc1), 2,3,9,10,16,17,23,24-octabutoxy (octyloxy)-29*H*,31*H*-phthalocyanine (Pc2), 2,9,16,23-tetra-*tert*-butyl-29*H*,31*H*-phthalocyanine (Pc3), 29*H*,31*H*-phthalocyanine (Pc4), 5,9,14,18,23,27,32,36-octabutoxy-2,3-naphthalocyanine (nPc1), 2,11,20,29-tetra-*tert*-butyl-2,3-naphthalocyanine (nPc2) and 2,3-naphthalocyanine (nPc3) as well as their synergistic interactions with I^−^ ions on the corrosion of aluminium in aqueous hydrochloric acid. The adsorption behaviour and corrosion inhibition potentials of these compounds in the absence and presence of I^−^ ions were investigated using a gravimetric method, electrochemical techniques and quantum chemical calculations. This study also reports how the nature and position of peripheral groups affect the aggregation of Pcs and nPcs and subsequently their corrosion inhibition properties. The molecular structures of the studied compounds are shown in Figure 1.

## 2. Results and Discussion

### 2.1. Gravimetric Measurements

Gravimetric measurements were carried out to determine the corrosion inhibition efficiency of the inhibitors at various concentrations and temperatures without and with addition of KI.

#### 2.1.1. Effect of Inhibitor Concentration and Temperature

**Figure 2:**
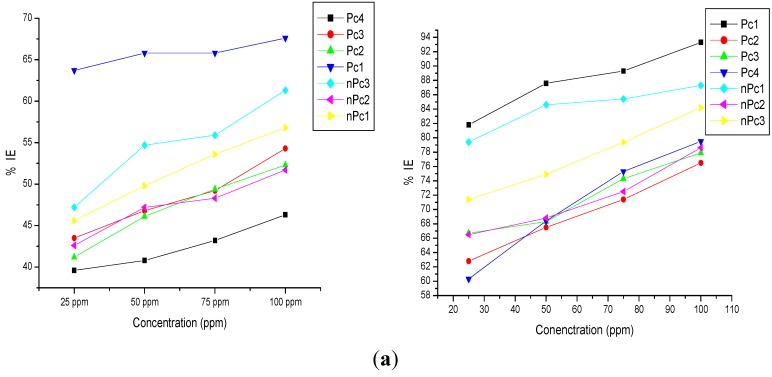

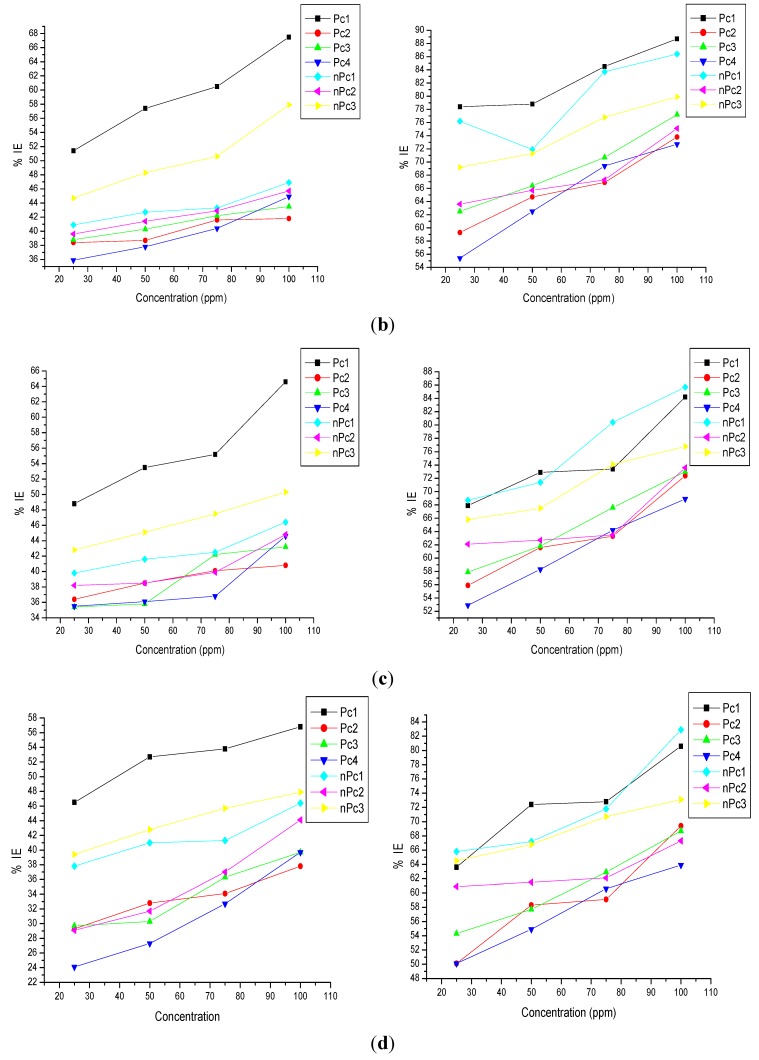

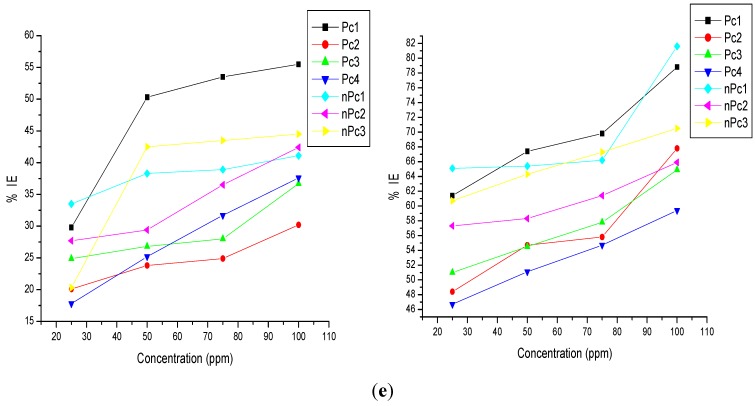
Inhibition efficiency *vs.* concentration of Pcs and nPcs without KI (**left hand side**) and with KI (**right hand side**) at (**a**) 303 K; (**b**) 313 K; (**c**) 323 K; (**d**) 333 K; and (**e**) 343 K.

The plots of inhibition efficiency, %IE against concentration for each inhibitor at different temperatures before and after the addition of potassium iodide (KI) are shown in Figure 2. The results show a general increase in %IE with increasing concentration of the inhibitors with Pc1 having the highest values of %IE over the range of concentrations studied. On the other hand, there is a general decrease in %IE with increase in temperature both without and with addition of KI. Among the nPc series, nPc3 has the highest values of %IE though not as high as Pc1. One obvious pattern in Figure 2 is the non-uniform trends of the %IE at higher concentrations, especially at 75 and 100 ppm. This is observed at all temperatures but more pronounced at higher temperatures. This observation may due to the varying aggregative interactions of the inhibitor molecules which has been reported to depend on solubility in the studied solvent and hence can be affected by concentrations and temperatures. This aggregation behaviour at higher concentrations and temperatures appears to favour the unsubstituted Pc4 more than the substituted Pc2 and Pc3. Hence, the %IE values of Pc4 at 100 ppm are higher than those of Pc2 and Pc3 at 40 °C–70 °C (Figure 2). This observation is not unexpected because, the aggregative interactions of phthalocyanines have been reported to depend on the chromophore, which in turn can be modified by the electronic and steric effects of the peripheral groups [27].

The lower %IE of Pc4 compared to other Pc compounds especially at low concentrations and temperatures can be attributed to the absence of electron donating alkyl or alkoxy substituents in Pc4 which however are present in Pc1, Pc2 and Pc3. The 1,4-butoxy substituents on Pc1 inform better electron donating ability and minimize steric hindrance compared to the 2,3-octaloxy substituents on Pc2. The closeness of the bulkier electron-donating octaloxy groups on Pc2 results in steric repulsion and hampers its inhibition potential compared to the Pc3 with relatively weaker but well-spaced electron-donating *tert*-butyl substituents.

Except at 70 °C, the %IE of the nPc-series compounds is in the order nPc3 > nPc1 > nPc2. The lower %IE of nPc1 with electron-donating butoxy substitutents compared to the unsubstituted nPc3 may be due to the competitive effects of steric hindrance and electron-donating features of the butoxy groups. The nPc3 with no substituents is also more likely to undergo aggregative interaction better than nPc1 and this could favour its corrosion inhibition potential.

Upon addition of KI, the %IE increased for each inhibitor which is an indication of synergism between the inhibitor molecules and I^−^ ions in solution. For instance, at 30 °C the %IE of Pc1 is 63.7% (at 25 ppm) without KI. This value was synergistically increased to 81.8% upon addition of KI. The %IE values in the presence KI are generally higher than in the absence of KI at all temperatures. The trend of %IE in the presence of KI is not entirely the same as in the absence of KI. This is due to different degree of interactions between I^−^ ions and the inhibitor molecules. For example in the nPc-series, nPc1 seems to exhibit better synergism with I^−^ ions than nPc2 and nPc3 possibly due to the presence of the relatively polar C-O bonds in its octabutoxy substituent groups which can bring about more enhanced interactions between its (nPc1) molecules and I^−^ ions. Despite its lower %IE without KI, nPc1 + KI shows higher %IE than nPc3 + KI. The %IE was found to decrease with increase in temperature both without and with addition of KI. This has been attributed to various reasons such as enhanced aggressiveness of the acid solution leading to rapid etching, the possible shift in the equilibrium position of adsorption/desorption process towards the desorption side, decomposition and/or rearrangement of the inhibitors at higher temperatures [33].

#### 2.1.2. Thermodynamic and Activation Parameters

A better understanding of the adsorption behaviour of an inhibitor can be obtained from the values of activation and thermodynamic adsorption parameters. The dependence of the corrosion rate on temperature can be expressed by Arrhenius equation:

(1) $$\log C_{R}=\log A-\frac{E_{a}}{2.303RT}$$

where *C_R_* is the corrosion rate (g·cm^−2^·h^−1^), E_a_ is the apparent activation energy, R is the molar gas constant (8.314 J·K^−1^·mol^−1^), T is the absolute temperature and A is the frequency factor. The values of the standard enthalpy and entropy of activation, ΔH* and ΔS* respectively were calculated from the transition state equation:

(2) $$C_{R}=\frac{RT}{Nh}\exp\left(\frac{\Delta S*}{R}\right)\exp\left(-\frac{\Delta H*}{RT}\right)$$

where *h* is Planck’s constant and *N* is the Avogadro number.

The Arrhenius plots of log *C_R_* *vs* 1/*T* for the corrosion of Al in 1 M HCl without and with various concentrations of the studied Pcs and nPcs are shown in Figure S1, while the slopes and standard deviations in the slopes of the obtained linear plots are listed in Table S1. The plots of log (*CR/T*) *vs.* 1/*T* (Figure S2) gave straight lines whose slope = −ΔH*/2.303*R* and intercept = log(*R/Nh*) + ΔS*/2.303*R*. The values of *E_a_* (obtained from the Arrhenius plots), ΔH* and ΔS* (obtained from the transition state equations) are listed in Table 1. The results in Table 1 show that the *E_a_* values in the presence of Pc2, Pc3 and Pc4 are generally higher than that of the blank acid, while the values of *E_a_* in the presence of nPc3 and Pc1 are lower than that of the blank. For nPc1 and nPc2, the *E_a_* values are lower than the *E_a_* value of the blank at higher concentrations of the inhibitors (70 and 100 ppm for nPc1; 100 ppm for nPc2). The positive values of ΔH* in Table 1 is an indication that the dissolution of Al and adsorption of the inhibitors are endothermic processes. Higher values of *E_a_* and ΔH* in the presence of an inhibitor have been attributed to physisorption mechanism, while lower values *E_a_* and ΔH* in the presence of an inhibitor have been attributed to chemisorption mechanism [34,35]. Various explanations have been provided in literature for a lower value of *E_a_* in the presence of an inhibitor compared to the acid blank. Lower *E_a_* values in the presence of inhibitor-contained system may be due to a slow rate of inhibitor adsorption with a resultant closer approach to equilibrium during the experiments at the higher temperature, or a shift of the net corrosion reaction from the region of the metal surface without adsorbed protective film to the covered region [36,37]. A more detailed discussion on various possibilities associated with lower values of *E_a_* in the presence of inhibitors was also provided by Bernali *et al.* [38]. The negative value of ΔS* in all cases indicates that the formation of the activated complex in the rate determining step is associative rather than dissociative and can be interpreted to decrease in disorderliness as the reaction proceeds from reactants to activated complex [39].

**Table 1:** Activation parameters derived from the Arrhenius plots without and with KI.

Without KI					With KI		
Inhibitor	Conc. (ppm)	*E_a_* (kJ·mol^−1^)	Δ*H* (kJ·mol^−1^)	Δ*S* (JK^−1^·mol^−1^)	*E_a_* (kJ·mol^−1^)	Δ*H* (kJ·mol^−1^)	Δ*S* (J·K^−1^·mol^−1^)
Blank	-	10.55	167.00	−275.79	10.55	167.00	−275.79
Pc1	25	8.68	137.38	−283.30	8.74	138.38	−277.32
	50	7.49	118.47	−287.40	7.70	121.86	−278.44
	75	7.70	121.79	−286.77	7.21	114.17	−275.74
	100	9.09	143.91	−284.52	8.77	138.76	−276.47
Pc2	25	11.41	180.62	−274.39	11.72	185.54	−273.75
	50	11.82	186.99	−273.21	12.38	195.97	−271.76
	75	11.68	184.80	−273.76	12.14	192.07	−272.68
	100	11.58	183.24	−274.13	12.08	191.25	−272.45
Pc3	25	11.50	181.92	−274.10	11.88	187.96	−273.22
	50	11.52	182.29	−274.08	11.71	185.26	−273.74
	75	11.73	185.60	−273.60	12.05	190.72	−272.89
	100	11.10	175.71	−275.68	11.65	184.43	−273.33
Pc4	25	11.70	185.22	−273.51	12.64	200.11	−270.91
	50	12.10	191.51	−272.39	12.33	195.18	−271.94
	75	11.94	188.97	−273.02	12.18	192.70	−272.54
	100	11.47	181.56	−274.66	12.26	194.04	−272.50
nPc1	25	10.39	164.37	−277.80	10.63	168.28	−277.32
	50	10.18	161.12	−278.56	10.31	163.18	−278.44
	75	10.74	169.99	−276.90	11.21	177.47	−275.74
	100	10.57	167.30	−277.66	11.05	174.94	−276.47
nPc2	25	11.11	175.76	−275.36	11.64	184.28	−273.90
	50	11.97	189.39	−272.82	12.31	194.75	−272.02
	75	11.40	180.42	−274.72	12.03	190.42	−273.09
	100	10.34	163.60	−278.29	10.92	172.76	−276.74
nPc3	25	7.97	126.09	−285.33	8.01	126.67	−285.49
	50	8.42	133.30	−284.17	8.66	133.30	−283.52
	75	8.31	131.49	−282.54	8.39	131.49	−282.27
	100	8.89	140.76	−282.86	9.18	140.76	−282.28

### 2.2. Adsorption Isotherms

**Figure 3:**
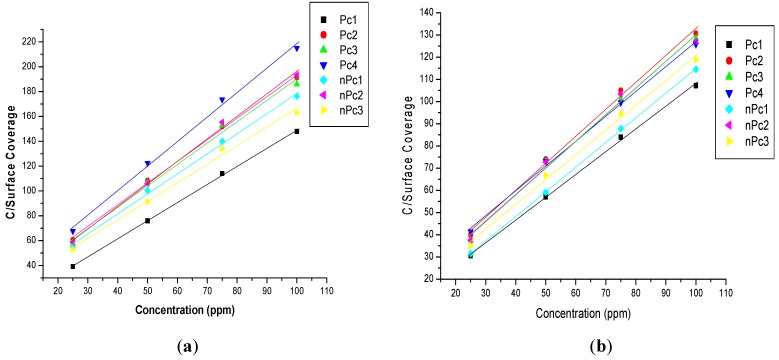
Langmuir adsorption isotherms (C/Surface Coverage *vs.* C) for the studied Pcs and nPcs (**a**) without KI, and (**b**) with KI at 303 K.

The onset of corrosion inhibition by organic compounds is often as a result of adsorption on metal surface. The adsorption behaviour of organic corrosion inhibitor on metal surface can be investigated by fitting the experimental data into suitable adsorption isotherms. Adsorption of inhibitor molecules on metal surface may occur via the displacement of water molecules from the active sites on the metal or meta/solution interface. This process may be represented by the reversible reaction equation:

Inh_(sol)_ + *x*H_2_O_(ads)_ ⇌ Inh_(ads)_ + *x*H_2_O_(sol)_ (3)

where *x* is the number of water molecules displaced by one molecule of the inhibitor. In order to gain more insight into the adsorption behaviour of the studied compounds, attempts were made to fit the experimental data into various adsorption isotherms including Langmuir, Frumkin, Freundlich and Temkin isotherms. But Langmuir isotherm gave the best fits with the highest near unity correlation coefficient (R^2^) values. According to the linear form of the Langmuir adsorption isotherm, the surface coverage, θ (θ = %IE/100) is related to the concentration of the inhibitor, *C_inh_* according to the equation:

(4) $$\frac{C_{inh}}{\text{θ}}=\frac{1}{K_{ads}}+C_{inh}$$

where *K_ads_* is the equilibrium adsorption constant. The plots of *C_inh_*/θ *vs.* *C_inh_* for all the seven compounds in the presence and absence of KI at 303 K are shown in Figure 3. Similar adsorption profiles were obtained at other temperatures but not reported in this study.

The change in free energy of adsorption (∆*G_ads_*) was calculated using the equation:

(5) $$\Delta G_{ads}=-RT\ln\left(55.5K_{ads}\right)$$

where R is the gas constant (8.314 J·K^−1^·mol^−1^), 55.5 is the molar concentration (mol·L^−1^) of water in the solution, *K_ads_* is the equilibrium constant for the adsorption process and T is the absolute temperature in Kelvin. The values of *K_ads_* and ∆*G_ads_* were calculated for all the studied compounds without and with addition of KI at various temperatures and the results are presented in Table 2. There is no regular pattern in the values of *K_ads_* obtained at various temperatures. However, the adsorption process is marked with large magnitude of *K_ads_* for all the studied inhibitors without and with KI (Table 2). This implies that adsorption of the inhibitors in the absence and presence of KI is a favourable process. The values of *∆G_ads_* for all the inhibitors without and with KI are negative, which implies that the adsorption process is spontaneous. The magnitude of ∆*G_ads_* can be used to predict the type of adsorption mechanism displayed by the inhibitors, that is, whether it is physisorption and/or chemisorption. A value of ∆*G_ads_* around −20 kJ·mol^−1^ or less negative has been attributed to electrostatic interactions between the charged inhibitor molecules and the charged metal surface (physisorption), while values around −40 kJ/mol or larger negative values involve charge sharing or charge transfer from organic molecules to the metal surface to form coordinate bond (chemisorption) [40]. The values of *∆G_ads_* (without KI) obtained in the present study are either less or slightly more negative than −20 kJ/mol, and it can be inferred from the results in Table 2 that for all the studied compounds (except nPc1) the adsorption process can be classified as physisorption especially at temperatures between 303–323 K. At higher temperatures (333–343 K) the values of *∆G_ads_* are slightly more negative than −20 kJ/mol suggesting that within this temperature range the adsorption of the studied compounds is essentially physisorption but involves competitive physisorption and chemisorption mechanisms. The adsorption of nPc1 (without KI) is physisorption at all temperatures. In the presence of KI, the values of *∆G_ads_* (Table 2) for all the studied inhibitors (except Pc2) are in the neighbourhood of −20 kJ/mol being slightly more negative. This again implies that the adsorption of these compounds upon synergistic interactions with KI is still mainly a physisorption process but also involves both physisorption and chemisorption mechanisms.

### 2.3. Potentiodynamic Polarization Measurements

**Figure 4:**
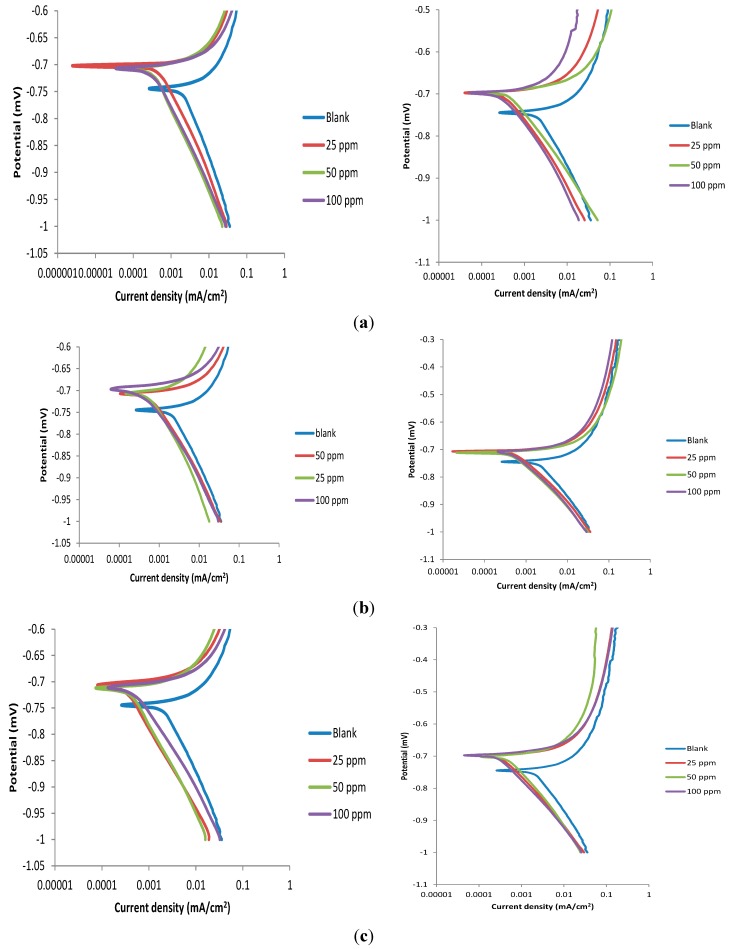

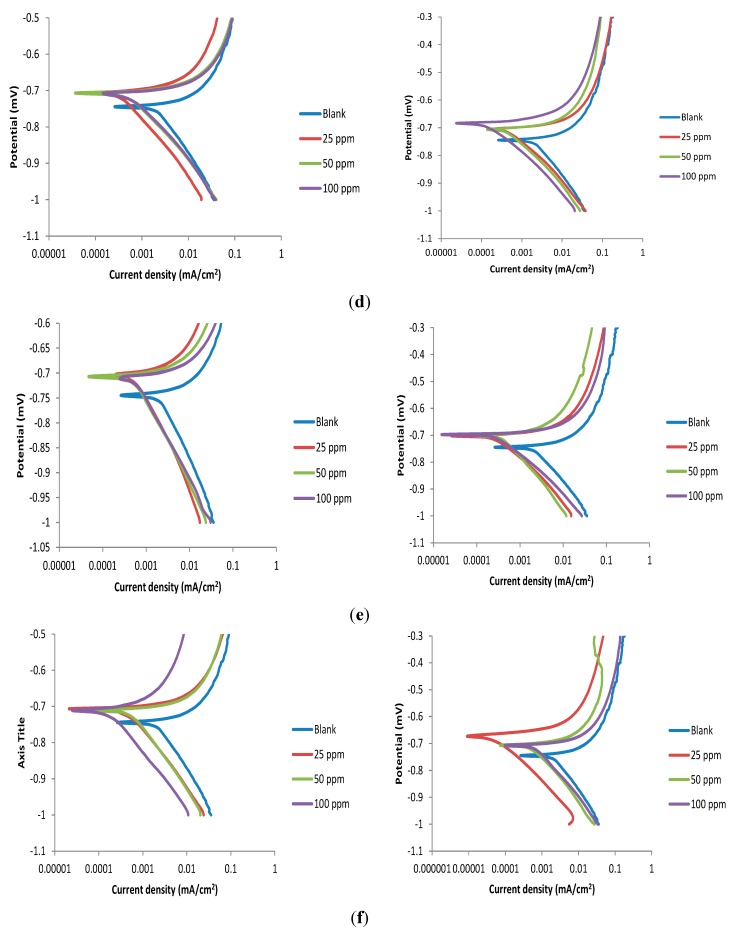

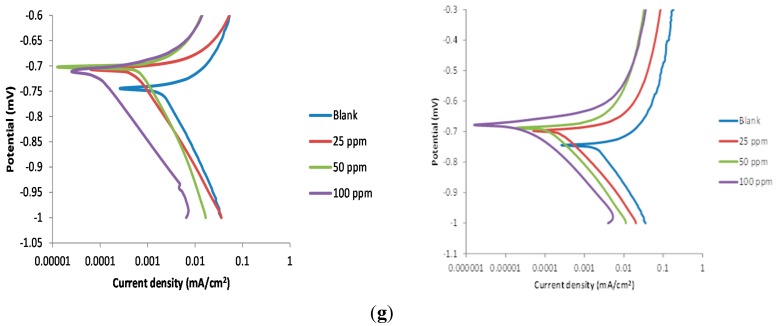
Potentiodynamic polarization curves for Al corrosion in 1 M HCl without and with difference concentrations of (**a**) Pc1; (**b**) Pc2; (**c**) Pc3; (**d**) Pc4; (**e**) nPc1; (**f**) nPc2; and (**g**) nPc3; without KI (**left**) and with KI (**right**).

The potentiodynamic polarization curves for Al in 1 M HCl without and with various concentrations of the studied Pcs and nPcs in the absence and presence of KI are shown in Figure 4. The polarization curves in Figure 4 shift to lower current region in the presence of the inhibitors without and with KI. This implies that the inhibitors reduce the corrosion current densities at the Al/electrolyte interface thereby reduce the rate of Al corrosion in 1 M HCl. The shapes of the polarization curves for the blank and inhibitor-containing electrochemical systems are similar, which suggests that the inhibitors retard Al corrosion in 1 M HCl by blocking the active sites on the Al surface without altering the mechanism of the corrosion reaction [9]. Electrochemical corrosion parameters such as corrosion potential (*E_corr_*), corrosion current density (*i_corr_*), anodic and cathodic Tafel slopes (*b_a_* and *b_c_* respectively) were obtained by the extrapolation of the Tafel regions of the polarization curves to the *E_corr_*. The percentage inhibition efficiency (%*IE_P_*) was calculated using the equation:

(6) $$\%IE_{P}=\frac{i_{corr}^{0}-i_{corr}^{i}}{i_{corr}^{0}}\times 100$$

where $i_{corr}^{0}$ and $i_{corr}^{i}$ are values of corrosion current density in the absence and presence of inhibitor respectively. The values of the *E_corr_*, *i_corr_*, *b_a_*, *b_c_* and %*IE_P_* are listed in Table 3.

**Table 2:** Thermodynamic parameters derived from the Langmuir adsorption isotherm for the studied Pcs and nPcs.

Without KI					With KI				
Inhibitor	T/K	*K_ads_* (10^3^ × mol^−1^)	−Δ*G°_ads_* (kJ∙mol^−1^)	Δ*H°* (kJ∙mol^−1^)	Δ*S°* (kJ∙mol^−1^)	*K_ads_* (10^3^ × mol^−1^)	−Δ*G°_ads_* (kJ∙mol^−1^)	Δ*H°* (kJ∙mol^−1^)	Δ*S°* (kJ·mol^−1^)
Pc1	303	22.99	−18.02	18.06	0.12	69.69	−20.81	21.56	2.46
	313	76.34	−21.04	21.08		84.75	−21.31	22.08	
	323	48.78	−19.91	19.95		85.47	−21.33	22.12	
	333	55.25	−20.23	20.27		136.99	−22.52	23.34	
	343	312.50	−24.59	24.64		180.18	−23.21	24.05	
Pc2	303	15.65	−17.05	17.08	0.09	42.28	−19.55	21.07	5.00
	313	34.25	−19.02	19.05		48.19	−19.88	21.45	
	323	90.09	−21.46	21.49		61.54	−20.50	22.11	
	333	99.01	−21.70	21.73		76.28	−21.04	22.70	
	343	51.68	−20.06	20.09		86.96	−21.37	23.08	
Pc3	303	17.41	−17.32	17.34	0.09	17.41	−20.27	23.08	9.27
	313	24.54	−18.18	18.21		24.54	−20.45	23.35	
	323	40.57	−19.45	19.48		40.57	−20.68	23.67	
	333	86.96	−21.37	21.40		86.96	−21.08	24.17	
	343	55.71	−20.25	20.28		55.71	−21.75	24.93	
Pc4	303	9.38	−15.76	15.79	0.11	54.95	−20.21	25.33	16.88
	313	14.42	−16.84	16.88		58.14	−20.36	25.64	
	323	35.03	−19.08	19.11		56.66	−20.29	25.74	
	333	40.98	−19.48	19.51		61.35	−20.49	26.11	
	343	46.84	−19.81	19.85		66.45	−20.69	26.48	
nPc1	303	54.94	−20.21	20.22	0.01	65.15	−20.64	19.45	−3.95
	313	51.68	−20.06	20.06		67.11	−20.72	19.48	
	323	64.72	−20.63	20.63		81.30	−21.20	19.93	
	333	76.63	−21.05	21.06		101.01	−21.75	20.43	
	343	59.00	−20.39	20.40		256.41	−24.10	22.74	
nPc2	303	17.41	−17.32	17.35	0.10	93.90	−21.56	28.91	24.25
	313	19.36	−17.59	17.62		134.23	−22.46	30.05	
	323	53.33	−20.14	20.17		82.99	−21.25	29.08	
	333	70.92	−20.86	20.89		92.59	−21.53	29.60	
	343	66.89	−20.71	20.74		96.62	−21.64	29.95	
nPc3	303	47.28	−19.84	19.86	0.09	108.70	−21.93	28.72	22.41
	313	51.95	−20.07	20.10		128.21	−22.35	29.36	
	323	20.41	−17.72	17.75		99.50	−21.71	28.95	
	333	116.96	−22.12	22.15		116.96	−22.12	29.58	
	343	173.91	−23.12	23.15		111.11	−21.99	29.67	

**Table 3:** Potentiodynamic polarization parameters such as corrosion potential (*E_corr_*), corrosion current density (*i_corr_*) and anodic and cathodic Tafel slopes (*b*_a_ and *b*_c_) using different inhibitors.

Without KI							With KI				
Inhibitor	Conc. (ppm)	*−E_corr_* (mV)	*i_corr_* (× 10^−3^) (µA)	*b_c_* (mV/dec)	*b_a_* (mV/dec)	% *IE_P_*	*−E_corr_* (mV)	*i_corr_* (× 10^−3^) (µA)	*b_c_* (mV/dec)	*b_a_* (mV/dec)	% *IE_P_*
Blank	-	742	1792	29	165	-	742	1792	29	165	-
Pc1	25	706	420	23	121	76.5	698	273	13	111	84.5
	50	707	334	20	167	81.4	695	393	14	128	84.8
	100	705	289	15	137	83.9	680	173	14	71	90.3
Pc2	25	707	532	34	154	70.3	705	409	11	110	77.2
	50	706	398	14	91	77.8	709	226	9	63	87.4
	100	695	269	18	100	85.0	708	405	17	124	77.4
Pc3	25	704	290	13	160	83.8	698	301	13	120	83.2
	50	710	332	12	139	81.5	700	421	2	137	76.5
	100	711	451	20	136	74.8	695	235	10	128	86.9
Pc4	25	437	832	78	79	83.0	704	398	17	115	77.8
	50	735	1209	24	146	76.9	705	373	13	131	79.2
	100	731	1031	21	147	74.2	684	152	24	117	91.5
nPc1	25	709	101	51	200	70.0	700	221	17	119	87.7
	50	705	294	11	138	86.1	699	324	33	142	81.9
	100	704	368	18	136	71.2	698	253	17	120	85.9
nPc2	25	704	280	10	104	84.3	671	17	11	46	99.1
	50	709	266	12	58	85.1	705	305	12	85	82.1
	100	707	158	29	147	91.2	707	506	13	125	71.0
nPc3	25	704	496	15	165	72.1	456	780	38	85	56.5
	50	701	613	29	105	65.3	685	116	19	124	93.5
	100	708	681	13	105	96.2	676	8	16	42	99.6

As reflected in Figure 4, the results in Table 3 also show that the polarization curves in the presence of the inhibitors shifted to more positive potentials (anodic region) compared to the blank, which suggests the formation of protective film of the inhibitors on the Al surface and reduction in the rate of anodic dissolution of the metal [41]. The magnitude of the displacement in *E_corr_* between the inhibitor-containing system and the blank can be used to classify the mode of inhibition into anodic, cathodic or mixed-type. An inhibitor is considered as anodic or cathodic type when the shift in *E_corr_* is greater than 85 mV [42]. In the present study, the magnitude of the shift in the *E_corr_* before and after the addition of the inhibitors is less than 85 mV, which implies that the studied Pcs and nPcs are mixed type corrosion inhibitors both without and with addition of KI. In other words, the studied compounds inhibit both the anodic dissolution of Al and cathodic hydrogen ion reduction. The relative magnitude of the change in the values of *b_a_* and *b_c_* before and after the addition of inhibitor has also been used in addition to the displacement in *E_corr_* to further classify the mode of inhibition as predominantly anodic or cathodic [42,43]. As shown in Table 3, the change in *b_a_* values between the inhibitor-containing systems and the blank are higher than that of *b_c_*, which indicates that the inhibitors predominantly inhibit anodic dissolution of Al, even though cathodic reaction is also inhibited. The *i_corr_* values in the presence of inhibitors are generally lower than that of the blank, which is an indication of corrosion inhibition effect of the studied compounds. Although a general conclusion cannot be made about the trend of %*IE_P_* with increasing concentration of the inhibitors, it is apparent from the results in Table 3 that all the studied compounds showed appreciable %*IE_P_* values. The non-uniform trend of %*IE_P_* with increasing concentration may be partly due to the reduced solubility of the Pcs and nPcs, which may lead to lower %*IE_P_* at the supposed higher concentrations. Lower %*IE_P_* at higher concentrations can also be as a result of desorption of the inhibitors from the initially blocked active sites on the metal surface. The differences in the values of %IE and %*IE_P_* from weight loss and electrochemical experiments respectively are not unexpected and could be attributed to the fact that weight loss measurement determines corrosion rate chemically, independent of the electrode potential, whereas potentiodynamic polarization measurements depend on the operational potential [44] and solubility plays a very important role in in this regards.

### 2.4. Synergism Consideration

The synergism parameter, *S_I_* was evaluated using the relationship given by Aramaki and Hackerman and also reported elsewhere [45,46,47,48] as:

(7) $$S_{I}=\frac{1-I_{1+2}}{1-I_{1+2}^{\prime }}$$

where *I*_1+2_ = *I*_1_ + *I*_2_; *I*_1_ = inhibition efficiency of the KI alone; *I*_2_ = inhibition efficiency of the Pc or nPc used as inhibitors (without KI) and *I′*_1+2_ = measured inhibition efficiency of Pc or nPc in combination with KI.

This parameter was evaluated from the inhibition efficiency values obtained from both the weight loss and polarization techniques and the results are presented in Table 4. The magnitude of *S_I_* can be used to deduce the nature of interaction, *i.e.*, synergistic or antagonistic that exists between I^−^ ions and the inhibitor molecules. It has been reported that a value of *S_I_* > 1 signifies synergistic effect while *S_I_* < 1 means antagonistic effect [49,50]. The *S_I_* values obtained from both methods as shown in Table 4 for different concentrations of the studied inhibitors at 303 K are greater than unity. This indicates that the improved inhibition efficiency caused by the addition of KI to the Pcs and nPcs is only due to synergistic effect.

The I^−^ ions can adsorb on the Al surface via strong chemisorption and then the synergistic interactions will take place between the adsorbed I^−^ ions and the cationic form of the inhibitor. The adsorption of the cationic form of the inhibitor on the Al surface by columbic attraction is facilitated by I^−^ ions that have already adsorbed on the Al surface by chemisorption. Stabilization of the adsorbed I^−^ ions with cations leads to a greater surface coverage and therefore greater inhibition. It could therefore be concluded that the addition of KI enhances the inhibition efficiency to a considerable extent due to the increase in the surface coverage in the presence of I^−^ ions.

**Table 4:** Synergistic parameters for the studied Pcs and nPcs with I^−^ ions calculated from weight loss and polarization experimental data.

	Synergistic Parameter (*S_I_*)		
Inhibitor	25 ppm	50 ppm	100 ppm
Pc1	1.42 (1.53)	1.35 (1.58)	1.28 (1.51)
Pc2	1.49 (1.59)	1.46 (1.49)	1.37 (1.78)
Pc3	1.44 (1.64)	1.45 (1.75)	1.37 (1.46)
Pc4	1.53 (1.74)	1.36 (1.64)	1.24 (1.38)
nPc1	1.23 (1.40)	1.21 (1.70)	1.25 (1.44)
nPc2	1.43 (1.38)	1.45 (1.68)	1.32 (2.03)
nPc3	1.24 (2.22)	1.21 (1.26)	1.25 (1.49)

( ) = Synergistic parameters from potentiodynamic polarization measurements.

### 2.5. Quantum Chemical Calculations

**Figure 5:**
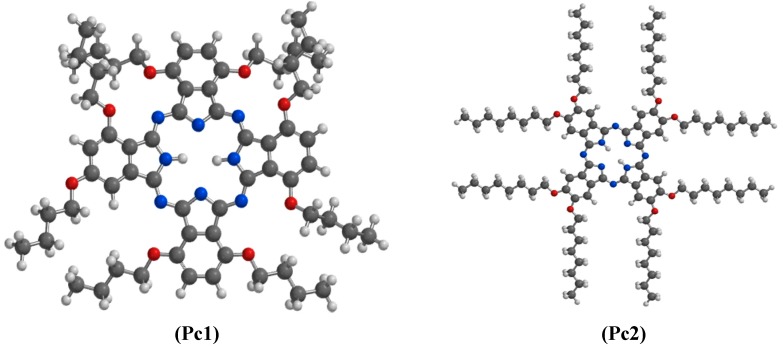

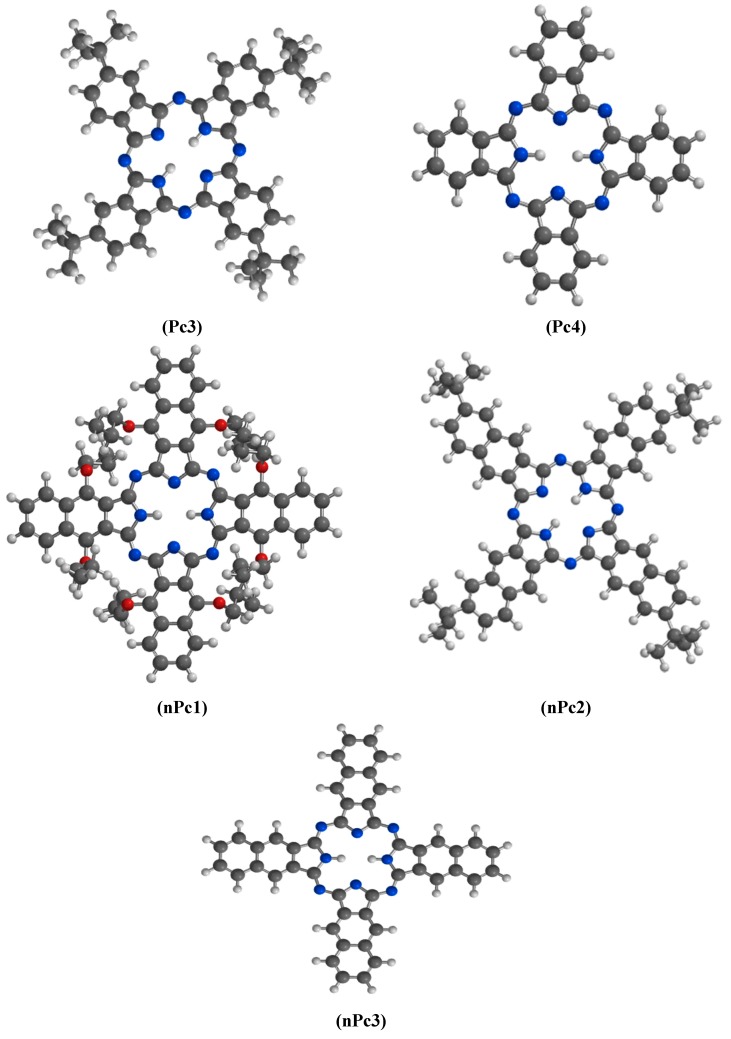
Optimized structures of the studied Pcs and nPcs. N = blue; O = red; H = white, and C = grey colour.

**Figure 6:**
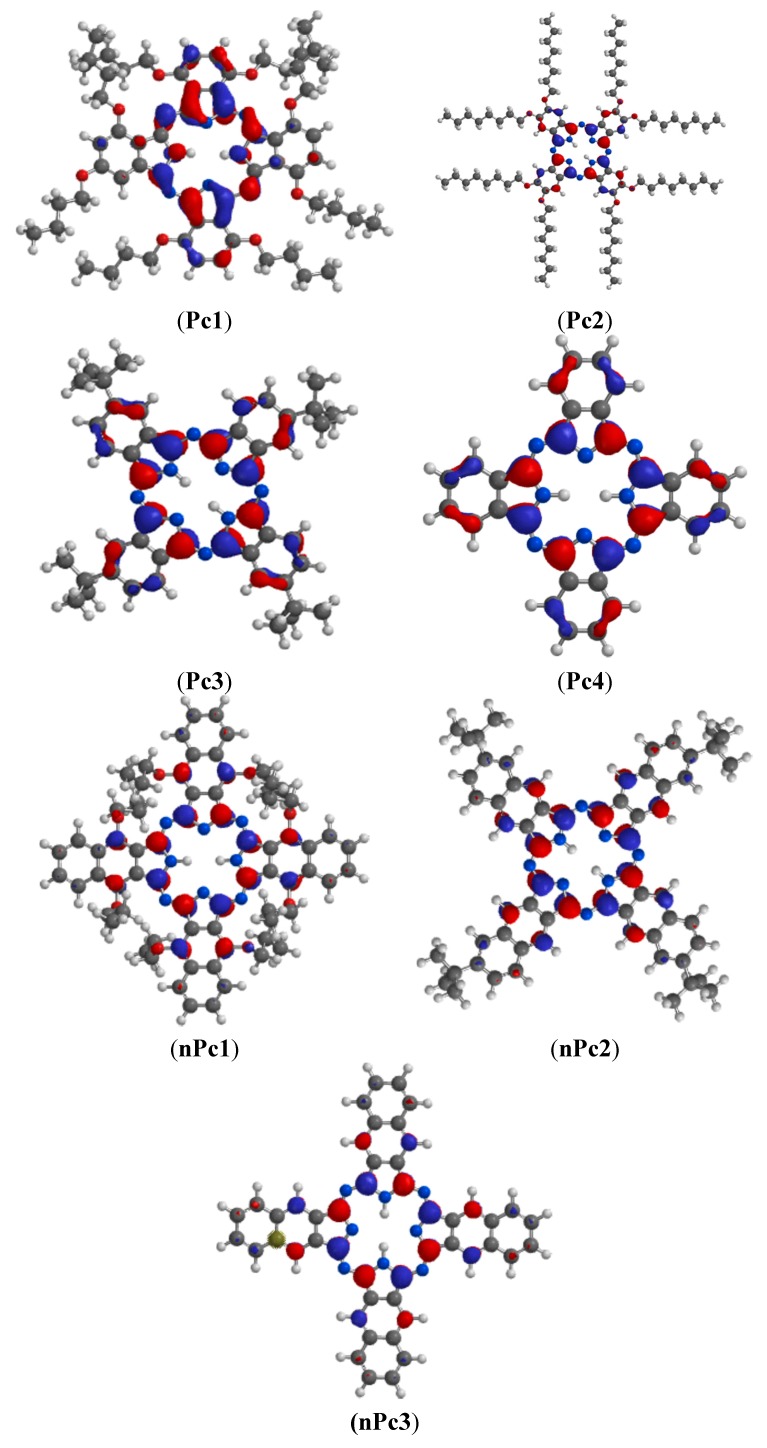
HOMO electron density distributions for the studied Pcs and nPcs.

**Figure 7:**
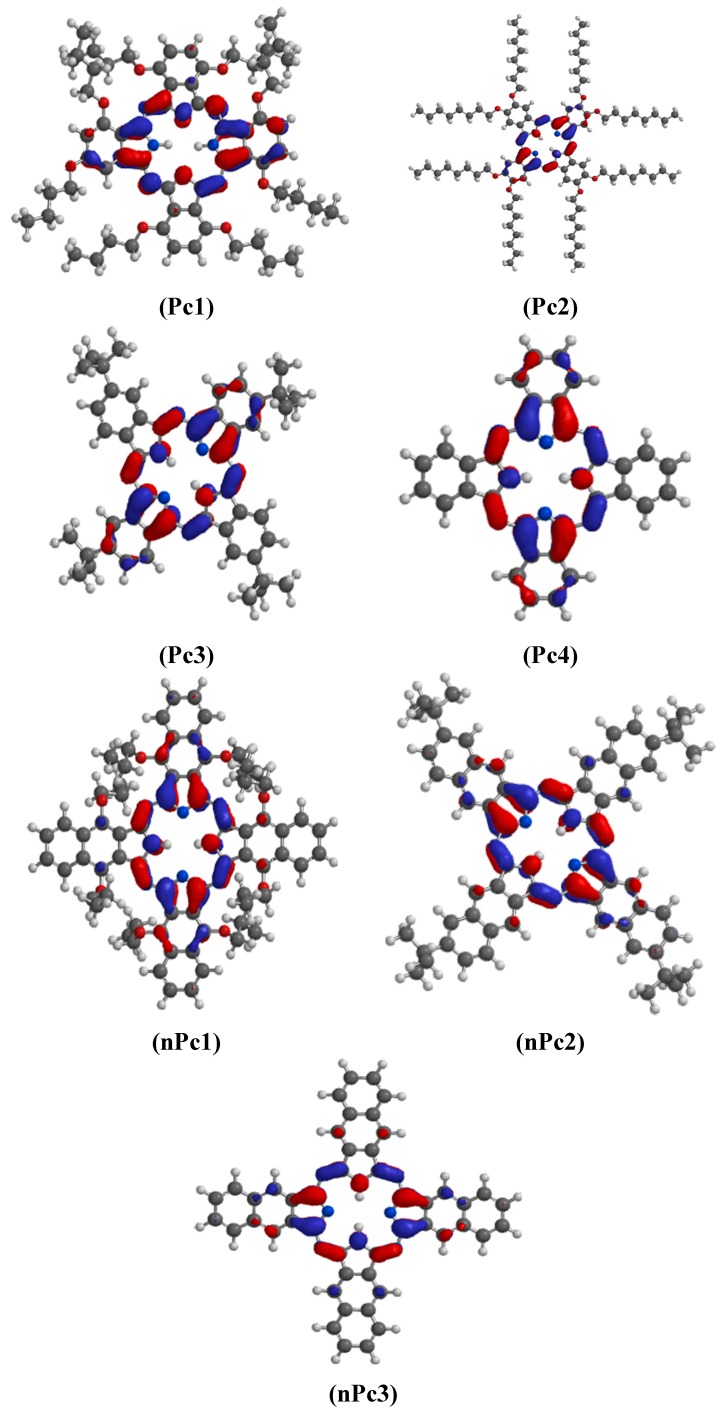
LUMO electron density distributions for the studied Pcs and nPcs.

The gas phase optimized geometries of the studied Pcs and nPcs are shown in Figure 5. The optimized geometries were confirmed to correspond to energy minima by the absence of imaginary frequencies in the vibrational frequency calculations. Selected optimized geometry parameters including the bond lengths, bond angles, dihedral angles, and the symmetry point groups of the studied Pcs and nPcs are listed in Table S1. The peripheral substituents did not have significant effect on the major bond lengths around the core of the molecules. The substituted Pcs and nPcs could not exhibit the supposed high symmetry due their complex molecular structures and repulsion between the bulky peripheral substituents, even though most of the dihedral angles at the core of the molecules are almost zero. Only the un-substituted Pc (Pc4) and nPc (nPc3) showed high symmetries (Table S2). All quantum chemical parameters used in comparison with experimental results are those derived from the ground state optimized geometries of the studied compounds. The frontier molecular orbitals (FMO) play important roles in informing the reactive sites of the inhibitors. According to the FMO theory, chemical reactivity largely depends on the highest occupied molecular orbitals (HOMO) and lowest unoccupied molecular orbitals (LUMO) of the interacting species. The HOMO and LUMO electron density surfaces of the studied Pcs and nPcs are shown in Figure 6 and Figure 7, respectively.

As shown in Figure 6, the HOMO of Pc1 comprises essentially π-orbitals together with few σ-type orbitals and delocalized mainly on the pyrrole ring and the nearby meso N atoms. This implies that Pc1 can interact with a metal atom by donating its HOMO π- or σ-orbital electrons to the respective low-lying vacant *d*- or *p*-orbitals of the metal. The HOMO of Pc2 is mostly distributed near the N and O atoms and is mainly of σ-type, suggesting that Pc2 will preferably interact with the low-lying vacant *p*-orbitals of Al. In Pc3 and Pc4, the HOMO orbitals comprise both π- and σ-type orbitals and are localized near the N atoms. The HOMO electron densities in nPc1, nPc2 and nPc3 are significantly σ-type orbitals and mostly distributed near the N atoms. This suggests that the nPc compounds can interact with the low-lying vacant *p*-orbitals of Al for the purpose of inhibiting Al corrosion.

As shown in Figure 7, the LUMO of Pc1 comprises a mixture of π- and σ-type orbitals, delocalized on the N atoms of the pyrrole rings, the linking imine N atoms, and extended to some C atoms of the condensed phenyl rings. This suggests that Pc1 can receive electrons from π- and/or σ-orbitals of Al during retro-donation. For Pc2, Pc3, and Pc4, the LUMO is delocalized on the N atoms of the pyrrole rings and the linking imine N atoms with mainly π-character and few σ-orbitals. For nPc1, nPc2 and nPc3, the LUMO electron densities are also distributed on the N atoms of the pyrrole rings, slightly extended to some C atoms of the condensed naphthalene rings and are essentially π-type.

Various chemical reactivity parameters were calculated for the studied compounds and are listed in Table 5. They include the energy of the HOMO (E_HOMO_), the energy of the LUMO (E_LUMO_), absolute hardness (η), absolute softness (σ) and dipole moment. The experimental inhibition efficiency values are also included in the table for easy comparison. The chemical reactivity parameters are discussed in two series such that the Pcs are compared separately and the nPcs also are compared separately. 

**Table 5:** Calculated quantum chemical parameters for the studied Pcs and nPcs.

	Reactivity Parameters								
Compounds	E_HOMO_	E_LUMO_	∆*E*	η	σ	∆*N*	ω	μ	%IE ^a^
Pcs									
Pc1	−4.11	−2.20	1.91	0.955	1.05	−2.01	5.21	1.09	67.6 (93.3)
Pc2	−4.44	−2.27	2.17	1.085	0.92	−1.68	5.19	0.03	52.3 (76.5)
Pc3	−4.80	−2.67	2.13	1.065	0.94	−1.53	6.55	0.16	54.3 (77.9)
Pc4	−4.98	−2.83	2.15	1.075	0.93	−1.44	7.09	0.01	46.2 (79.5)
**nPcs**									
nPc1	−4.25	−2.58	1.67	0.835	1.20	−2.15	6.98	0.09	56.8 (87.3)
nPc2	−4.43	−2.46	1.97	0.985	1.02	−1.80	6.02	0.00	51.7 (78.6)
nPc3	−4.55	−2.76	1.79	0.895	1.12	−1.87	7.46	0.00	61.3 (82.5)

^a^ percentage inhibition efficiency at 303 K for 100 ppm of the studied compounds obtained from weight loss measurements both without and (with KI).

The trend of the E_HOMO_ for the studied Pcs is Pc1 > Pc2 > Pc3 > Pc4. This suggests that Pc1 has the highest tendency to donate electrons to the appropriate low-lying empty or partially filled orbitals of Al atom, while Pc4 has the least tendency to donate electrons to the metal. The effect of peripheral/substituent groups on the electron-donating ability of Pcs is apparent in the trend of the E_HOMO_ because, Pc1, Pc2 and Pc3 with electron-donating ether or alkyl substituent groups exhibit higher E_HOMO_ than the unsubstituted Pc4. The Pc1 and Pc2 have ether substituents, while Pc3 has alkyl substituents. The higher E_HOMO_ values of Pc1 and Pc2 compared to Pc3 is due to better electron-donating ability of the ether group than the alkyl group. The longer chain ether/alkyl group is generally a better electron-donor than the shorter chain member of the same group. It is therefore expected that the Pc2 with the longer -O-(CH_2_)_7_CH_3_ substituents will have higher value of E_HOMO_ compared to the Pc1 with the shorter -O-(CH_2_)_3_CH_3_ substituents. However, the reverse trend was observed, which may be due to the difference in the substituent positions. The overall electron-donating effect of the shorter chain -O-(CH_2_)_3_CH_3_ groups that are in para positions relative to each other is higher than that of the longer chain -O-(CH_2_)_7_CH_3_ groups that are in ortho positions relative to each other. The trend of the E_LUMO_ for the studied Pc compounds is Pc1 > Pc2 > Pc3 > Pc4, suggesting that Pc4 has the highest tendency to accept electrons from the appropriated occupied orbitals of the Al atom. The trend of the E_LUMO_ values is also in line with the observed results for the E_HOMO_ and the same explanations apply.

The trend of the values of ΔE for the Pcs is Pc2 > Pc4 > Pc3 > Pc1, which suggests that Pc1 is the most reactive species while Pc2 is the least reactive. The trends of E_HOMO_, E_LUMO_ and ΔE are in support of the highest %IE of Pc1 obtained from the weight loss measurements. Though, the trends cannot be generalized for all the studied Pcs at different concentrations and temperatures. The trend of the dipole moment, μ for the Pcs is Pc1 > Pc3 > Pc2 > Pc4, which is in agreement with the %IE listed in Table 5. The trend of the E_HOMO_ for the studied nPcs is nPc1 > nPc2 > nPc3, which is directly related to the nature and positions of the electron-donating substituents on the nPcs as previously explained above for the Pcs. The nPc1 with the better electron-donating ether substituents has the highest tendency to donate electrons to the appropriate vacant low-lying orbitals of Al atom, while the unsubstituted nPc3 has the least tendency to donate electrons to the metal atom. The decreasing order of the E_LUMO_ is nPc3 > nPc1 > nPc2, suggesting that nPc3 has the highest tendency to accept electrons from the occupied orbitals of Al atom in retro-donation step. The trend of E_LUMO_ is in good agreement with the order of the experimental %IE (Table 5). The trend of ΔE values for the nPcs is nPc2 > nPc3 > nPc1, which portrays nPc1 as the most reactive compound with the highest tendency to interact with the metal surface among the studied nPcs. This trend however is not the same as the order of the experiment %*IE* (Table 5).

Since Pc1 and nPc1 have the same peripheral/substituent groups at similar positions on the rings, the two compounds can be compared. The results in Table 5 show that Pc1 is a better corrosion inhibitor than nPc1. This inference is supported by the %IE values listed in Table 5 and also the values of E_HOMO_ and µ. Similarly, Pc3 can be compared with nPc2 and the values of E_LUMO_, ω and µ support the higher %IE of Pc3 compared to nPc2. A comparison of the unsubstituted compounds Pc and nPc (Pc4 and nPc3) show that the lower ∆E, and higher E_HOMO_, σ, and ω values of nPc3 are in support of its higher %IE compared to Pc4. This implies that naphthalocyanine is a better corrosion inhibitor than phthalocyanine without peripheral/substituent groups. This is due to the presence of naphthalene rings in nPc, making it to have more π-electrons and aromatic rings. However, the trend changes in the presence of substituent groups on the two compounds, which may be attributed to various reasons including change in the degree of aggregative interactions and solubility properties.

The Mulliken atomic charges on non-hydrogen atoms of the studied Pc and nPc compounds are reported in Tables S3 and S4 (Supplementary Materials) respectively. In Pc1, the highest negative charges are found on the N atoms followed by the O atoms. This implies that the N and O atoms are the most likely atoms to donate electrons to the empty/partially filled *d* or *p* orbitals of the metal. The negative charges on the N atoms follow the order N1 > N5 > N7 which suggests that the N1 atom is the most preferred site for σ ↔ *p* orbitals interactions, while N5 will be preferred for π ↔ *d* orbitals interactions. Similar results as found for Pc1 were also obtained for Pc2, Pc3 and Pc4 and the trends of the negative Mulliken charges on the atoms are:

Pc2: N3 > N1 > N5; Pc3 and Pc4: N1 > N5 > N3

such that the preferred sites of interactions with the metal atom are:

Pc2: N3 (π ↔ *d*) and N1 (σ ↔ *p*); Pc3 and Pc4: N1 (σ ↔ *p*) and N5 (π ↔ *d*)

For the nPc series of the studied compounds, the highest negative Mulliken atomic charge is the N atoms followed by the O atoms for the nPc1, nPc2 and nPc3 and the order of the magnitude of the negative charge is N1 > N5 > N3, indicating that N1 would have the highest tendency to donate lone pair of electrons to the available low-lying *p*-orbitals of the metal atom, while N5 would have the highest tendency to donate lone pair of electrons to the available low-lying *d*-orbitals of the metal atom. 

A comparison of Pc1 and nPc1 suggests that the negative charge on the N atoms is higher on nPc1 than Pc1. This means that nPc1 would have greater tendency to donate electrons to the metal surface. The high negative charge on the N atoms of the nPc1 is mainly due to the electrons delocalization on the naphthalocyanine as a result of the resonance nature of the ring. The presence of extra aromatic rings in the nPc1, as compared to Pc1, increases the electron density in the nPc ring, which in turn increases the negative charge on the N atoms.

The condensed Fukui functions (*f^+^* and *f^−^*) are used to assess the susceptibility of individual atomic sites in a molecule to nucleophilic or electrophilic attacks. The nucleophilic Fukui function, *f*^+^ measures the change in electron density at various sites when a molecule gains electrons and it corresponds to the reactivity of the sites with respect to nucleophilic attack. On the other hand, the electrophilic Fukui function, *f*^−^ corresponds to reactivity with respect to electrophilic attack when the molecule loses electrons [51,52]. The values of *f*^+^ and *f*^−^ for the studied Pcs and nPcs are listed in Tables S5 and S6 respectively. From the results in Table S5, C_1a_ (Pc1), C_4_ (Pc2), N_5_ (Pc3), N_5_ & N_3_ (Pc4), N_5_ (nPc1), C_13a_ (nPc2) and N_5_ & N_13_ have the highest value of *f*^+^, suggesting that these sites are the most susceptible to nucleophilic attacks, while, N1 (Pc1), N3 (Pc2), N5 (Pc3), C1e (Pc4), C1a (nPc1), C13j (nPc2) and C13a have the highest value of *f*^−^, suggesting that these atoms are the most preferred sites for electrophilic attacks.

### 2.6. Quantitative Structure Activity Relationship (QSAR)

The correlation of individual quantum chemical parameters with the inhibition efficiency of the inhibitors is usually less informative because of the complexity of the adsorption process. It is therefore essential to combine several quantum chemical parameters to form a composite index that could be correlated to the experimental inhibition efficiency (%IE). A correlation between quantum chemical parameters and the observed %IE is studied by means of quantitative structure activity relationship (QSAR) approach in which relevant mathematical equations are used to relate the quantum chemical parameters to the observed inhibition efficiency of an inhibitor. The derived equations are used to predict %IE from the concentrations of the inhibitors and to provide theoretical explanations for the effects of different variables studied [53,54]. In the present work, two models were tested; the linear model and the non-linear models proposed by Lukovits *et al.* for the study of interactions of corrosion inhibitors with metal surface in acidic solutions [53,54]. However, only the linear model produced the best correlation results between experimental and theoretical data. This linear equation is of the form:

IE_theor_ = Ax_i_ C_i_ + B (8)

where A and B are the regression coefficients determined through regression analysis, x_i_ is a quantum chemical index characteristic of the molecule i, C_i_ is the experimental concentration of the inhibitor.

QSAR was performed using the quantum chemical parameters obtained from the B3LYP/6-31G (d,p) method and those obtained using the AM1 and PM3 methods in an attempt to correlate quantum chemical parameters obtained from different methods with the observed inhibition efficiency. The results of the QSAR analysis of the quantum chemical parameters obtained with B3LYP/6-31G(d,p) method produced the best correlation, which showed that a combination of two quantum chemical parameters to form a composite index provides the best correlation with the experimental data and the best equations obtained are:

IE = 0.553 × E_HOMO_ × C_i_ − 0.457 × E_LUMO_ × C_i_ + 194.786 R^2^ = 0.938 and SSE = 15.06

IE = −0.332 × ∆E × C_i_ − 0.197 × ∆N × C_i_ + 91.578   R^2^ = 0.934 and SSE = 16.06

where R^2^ is the coefficient of determination, and SSE is the sum of squared errors defined as:

(9) $$\text{SSE}={\sum \left(I{\%}_{\exp erimental}-I{\%}_{theoretical}\right)}^{2}$$

**Figure 8:**
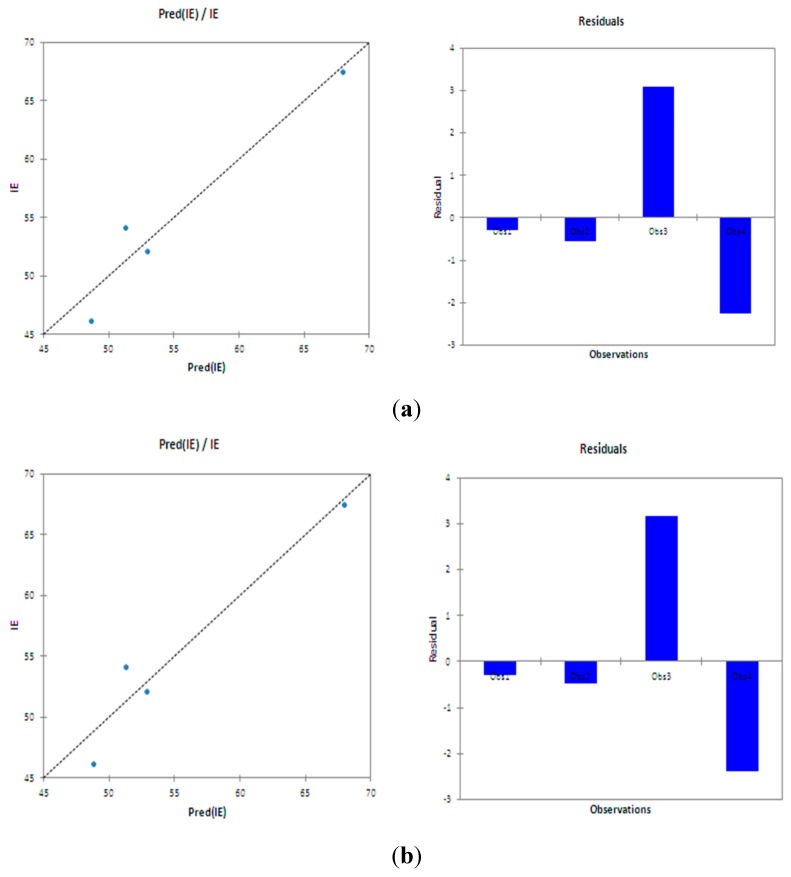
Representative plots of correlation between the theoretically estimated %IE and experimentally obtained %IE using (**a**) E_HOMO_ and E_LUMO_ energies and (**b**) Δ*E* and Δ*N*.

The first equation suggests that a higher E_HOMO_ and lower E_LUMO_ results in greater inhibition efficiency; the second equation suggests that the smaller ∆E and ∆N of an inhibitor, the greater the inhibition efficiency of the inhibitor. The corresponding representative plots of the correlation between experimental inhibition efficiency and theoretically estimated inhibition efficiency are shown in Figure 8.

## 3. Experimental Section

### 3.1. Materials and Aggressive Solutions

The phthalocyanines and naphthalocyanines used as corrosion inhibitors were obtained commercially from Sigma-Aldrich Chemicals (Aston Manor, South Africa). Hydrochloric acid, tetrahydrofuran (THF), acetone and potassium iodide (KI) were obtained from Merck Chemicals (Modderfontein, South Africa). All the reagents and solvents were used without further purification.

Corrosion tests were performed on 100% Al sheets with freshly prepared surface. The surface of the Al specimens were ground using silicon carbide papers of various grades (600–1000), washed with distilled water, degreased in acetone, wiped with a clean towel paper and finally air-dried.

Aggressive solution of 1 M HCl was prepared by diluting 32% analytical grade using distilled water. Phthalocyanines and naphthalocyanines were first dissolved in minimum amount (10 mL equivalent to 4% by vol.) of tetrahydrofuran (THF) and later diluted to various concentrations (25, 50, 75 and 100 ppm) in 250 mL volumetric flask. A fixed concentration (0.5 M) of KI was prepared and used for the synergistic studies.

### 3.2. Gravimetric Method

Al sheets of the dimension 1 cm × 8 cm were used for the gravimetric experiments. Surface pre-treatment was carried out as described in Section 3.1 above. The samples were then weighed (w_1_) and suspended freely in glass reaction vessels with the aid of glass hooks and rods. The suspended samples were completely immersed in 100 mL of the aggressive solutions of 1 M HCl without and with various concentrations of the studied inhibitors at 303–343 K in thermostated water baths. The samples were retrieved after 12 h, gently brushed, washed with distilled water, rinsed with acetone, dried with warm air and finally re-weighed (w_2_). The experiment was conducted in triplicates for each concentration and the average weight loss (W = (w_1_ − w_2_)/3 in grams) was recorded.

The corrosion rate (ρ in gcm^−2^·h^−1^), percentage inhibition efficiency (%IE) and surface coverage (θ) were calculated from the weight loss using the equations:

(10) $$\text{ρ}=\frac{W}{St}$$

(11) $$\%IE=\left(\frac{{\text{ρ}}_{1}-{\text{ρ}}_{2}}{{\text{ρ}}_{1}}\right)\times 100$$

(12) $$\text{θ}=\left(\frac{{\text{ρ}}_{1}-{\text{ρ}}_{2}}{{\text{ρ}}_{1}}\right)$$

where *W* is the average weight loss of the Al sheets, *S* is the total surface area of the Al specimen (cm^2^), *t* is the immersion time (h), while ρ_1_ and ρ_2_ are the corrosion rates without and with inhibitors respectively.

### 3.3. Electrochemical Measurements

Al sheets of the dimension 1 cm × 1 cm were used for all the electrochemical studies. Surface pre-treatment was carried out as described in the previous section. All electrochemical measurements were carried out on Al samples with the exposed area of 1 cm^2^. The conventional three electrode electrochemical cell system was used with Ag/AgCl, 3 M KCl as the reference electrode, platinum rod as the counter electrode and the Al coupon with freshly prepared surface of 1 cm^2^ exposed surface area as the working electrode. Potentiodynamic polarization measurements were carried out at a scan rate of 2 mV/s. All electrochemical measurements were carried out at room temperature (±25 °C) using the Autolab potentiostat/galvanostat, PGSTAT302 N (Ecochemie, Utrecht, The Netherlands). The equipment is controlled by the general purpose electrochemical software (GPES) version 4. Specimens were immersed in the test solutions for 1 h to allow for a stable open circuit potential (OCP) before each electrochemical measurement. 

Electrochemical parameters such as the corrosion current density (*i_corr_*), anodic and cathodic Tafel slopes (*b_a_* and *b_c_* respectively) were obtained by extrapolating the Tafel regions of the polarization curves to the corrosion potential (*E_corr_*). The percentage inhibition efficiency (%IE) was calculated from the equation:

(13) $$\%IE=\frac{i_{corr}^{0}-i_{corr}^{i}}{i_{corr}^{0}}\times 100$$

where $i_{corr}^{0}$ and $i_{corr}^{i}$ are the values of corrosion current density in absence and presence of inhibitor respectively.

### 3.4. Quantum Chemical Studies and Quantitative Structure Activity Relationship (QSAR)

Gas phase geometry optimizations and vibrational frequency calculations were carried out on the studied phthalocyanines and naphthalocyanines without symmetry constraint. The density functional theory (DFT) method using the Becke’s three parameter hybrid functional together with Lee-Yang-Parr correlation functional (B3LYP) was used in combination with the Pople type basis set 6-31G(d) for all the calculations [55,56,57,58]. The B3LYP functional has been widely used in literature in conjunction with different Pople’s basis sets to produce satisfactory geometries at relatively less computational cost [59,60,61,62]. The optimized structures were confirmed to correspond to true energy minima with the absence of imaginary frequency in the force constant calculations. The optimized structures and electron density surfaces were visualized using the Spartan 10 software program. The calculated parameters include the energy of the highest occupied molecular orbital (E_HOMO_), energy of the lowest unoccupied molecular orbital (E_LUMO_), energy gap (∆*E*), dipole moment (μ), global softness (σ), global hardness (η), electrophilicity (ω), fraction of electrons transferred (∆N), electronegativity (χ), electron affinity (EA) and ionization potential (IE).

Electronegativity (χ) is the measure of the power of an electron or group of atoms to attract electrons towards itself and it can be estimated by using the equation:

χ ≅ −½ (E_HOMO_ + E_LUMO_) (14)

Global hardness (η) measures the resistance of an atom to a charge transfer and was estimated using the equation:

η ≅ −½ (E_HOMO_ − E_LUMO_) (15)

Global electrophilicity index (ω) was estimated by using the electronegativity and chemical hardness parameters through the equation:

ω = χ^2^/2η (16)

A high value of electrophilicity describes a good electrophile while a small value of elecrophilicity describes a good nucleophile.

Global softness (σ), describes the capacity of an atom or group of atoms to receive electrons [56], it was estimated by using the equation:

σ = 1/η ≅ −2/(E_HOMO_ − E_LUMO_) (17)

Electron affinity (A) is the energy released when an electron is added to a neutral molecule; it is related to E_LUMO_ through the equation:

A ≅ −E_LUMO_ (18)

Ionization potential (I) is the amount of energy required to remove an electron from a molecule; it is related to the energy of the E_HOMO_ through the equation:

I ≅ −E_HOMO_ (19)

The quantitative structure activity relationship (QSAR) is another powerful tool for correlating the quantum chemical parameters of inhibitors with the experimental inhibition efficiencies. The QSAR is versatile because it can be used to generate empirical equations comprising multiple inter-related quantum chemically derived molecular and/or electronic parameters that might be contributing to the efficiency of the studied corrosion inhibitors. The quantum chemical parameters of the studied molecules were fitted into both the linear and non-linear forms of the QSAR equations proposed by Lukovits *et al.* and the best equations were selected based on the correlation coefficient (R^2^) and sum of squared error (SSE) values [53,63]. The QSAR plots and the corresponding equations were derived with the aid of the XLSTAT program [64].

## 4. Conclusions

Seven macrocylic compounds comprising four phthalocyanines (Pcs) and three naphthalocyanines (nPcs) were studied for their inhibition potentials on Al corrosion in 1 M HCl using gravimetric method, potentiodynamic polarization technique, quantum chemical calculations and QSAR approach. Synergistic effects of I^−^ ions with these compounds were also investigated. The following conclusions can be drawn from the results:

(1) All the studied Pcs behave as good corrosion inhibitors for Al in 1 M HCl solution with Pc1 being the best inhibitor among the Pcs and nPc3 being the best among the nPcs.
(2) The addition of potassium iodide (KI) to the Pc and nPc solutions increases the inhibition efficiency.
(3) The adsorption of the studied compounds on Al surface obeys the Langmuir adsorption isotherm.
(4) The thermodynamic and kinetic parameters revealed that the adsorption of the studied compounds on Al surface is spontaneous and involves both physisorption and chemisorption mechanisms.
(5) The quantum chemical parameters showed that the studied Pcs and nPcs have the ability to donate/accept electrons to/from appropriate p and/or d orbitals of metal atoms and support their good corrosion inhibition potentials.
(6) The experimental results revealed the possibility of aggregative interactions between the inhibitor molecules and the results further indicated that these interactions are affected by the peripheral groups on the compounds.

## Supplementary Materials

Supplementary materials can be accessed at: http://www.mdpi.com/1420-3049/20/09/15701/s1.

## References

[1] Li X., Nie X., Wang L., Northwood D.O. (2005). Corrosion protection properties of anodic oxide coatings on an Al-Si alloy. Surf. Coat. Technol..

[2] Khaled K.F., Amin M.A. (2009). Electrochemical and molecular dynamics simulation studies on the corrosion inhibition of aluminum in molar hydrochloric acid using some imidazole derivatives. J. Appl. Electrochem..

[3] Arellanes-Lozada P., Olivares-Xometl O., Guzmán-Lucero D., Likhanova N.V., Domínguez-Aguilar M.A., Lijanova I.V., Arce-Estrada E. (2014). The inhibition of aluminium corrosion in sulfuric acid by poly(1-vinyl-3-alkyl-imidazolium hexafluorophosphate). Materials.

[4] Muniandy M.T., Rahim A.A., Osman H., Shah A.M., Yahya S., Raja P.B. (2011). Investigation of some schiff bases as corrosion inhibitors for aluminium alloy in 0.5 M hydrochloric acid solutions. Surf. Rev. Lett..

[5] Cabot P.L., Centellas F.A., Garrido J.A., Pérez E., Vidal H. (1991). Electrochemical study of aluminium corrosion in acid chloride solutions. Electrochim. Acta.

[6] Brett C.M.A. (1992). On the electrochemical behaviour of aluminium in acidic chloride solution. Corros. Sci..

[7] Sastri V.S. (1998). Corrosion Inhibitors: Principles and Applications.

[8] Bregman J.I. (1963). Corrosion Inhibitors.

[9] Olasunkanmi L.O., Obot I.B., Kabanda M.M., Ebenso E.E. (2015). Some quinoxalin-6-yl derivatives as corrosion inhibitors for mild steel in hydrochloric acid: Experimental and theoretical studies. J. Phys. Chem. C.

[10] Gajek A., Zakroczymski T., Romanchuk V., Topilnytsky P. (2012). Protective properties and spectral analysis of nitrogen- and oxygen-containing corrosion inhibitors for oil equipment. Chem. Chem. Technol..

[11] Guzman-Lucero D., Olivares-Xometl O., Martinez-Palou R., Likhanova N.V., Dominguez-Aguilar M.A., Garibay-Febles V. (2011). Synthesis of selected vinylimidazolium ionic liquids and their effectiveness as corrosion inhibitors for carbon steel in aqueous sulfuric acid. Ind. Eng. Chem. Res..

[12] Sherif E.M., Park S.M. (2006). Effects of 1,4-naphthoquinone on aluminum corrosion in 0.50 M sodium chloride solutions. Electrochim. Acta.

[13] Mazhar M.A., Badawy W.A., Abou Romia R.M. (1986). Impedance studies of corrosion resistance of aluminium in chloride media. Surf. Coat. Technol..

[14] Tomcsányi L., Varga K., Bartik I., Horányi H., Maleczki E. (1989). Electrochemical study of the pitting corrosion of aluminium and its alloys—II. Study of the interaction of chloride ions with a passive film on aluminium and initiation of pitting corrosion. Electrochim. Acta.

[15] Rio Y., Rodríguez-Morgade M.S., Torres T. (2008). Modulating the electronic properties of porphyrinoids: A voyage from the violet to the infrared regions of the electromagnetic spectrum. Org. Biomol. Chem..

[16] Martínez-Díaz M.V., de la Torre G., Torres T. (2010). Lighting porphyrins and phthalocyanines for molecular photovoltaics. Chem. Commun..

[17] Bottari G., Trukhina O., Ince M., Torres T. (2012). Towards artificial photosynthesis: Supramolecular, donor-acceptor, porphyrin and phthalocyanine/carbon nanostructure ensembles. Coord. Chem. Rev..

[18] Wöhrle D., Suvorova O., Gerdes R., Bartels O., Lapok L., Baziakina N., Makarov S., Slodek A. (2004). Efficient oxidations and photooxidations with molecular oxygen using metal phthalocyanines as catalysts and photocatalysts. J. Porphyr. Phthalocyanines.

[19] Nyokong T. (2007). Effects of substituents on the photochemical and photophysical properties of maingroup metal phthalocyanines. Coord. Chem. Rev..

[20] Zagal J.H., Griveau S., Silva J.F., Nyokong T., Bedioui F. (2010). Metallophthalocyanine-based molecular materials as catalysts for electrochemical reactions. Coord. Chem. Rev..

[21] Aoki I.V., Guedes I.G., Maranhao S.L. (2002). Copper phthalocyanine as corrosion inhibitor for ASTM A606-4 steel in 16% hydrochloric acid. J. Appl. Electrochem..

[22] Zhao P., Liang Q., Li Y. (2005). Electrochemical, SEM/EDS and quantum chemical study of phthalocyanines as corrosion inhibitors for mild steel in 1 mol/L HCl. Appl. Surf. Sci..

[23] Feng Y., Chen S., Guo W., Liu G., Ma H., Wu L. (2007). Electrochemical and molecular simulation studies on the corrosion inhibition of 5,10,15,20-tetraphenylporphyrin adlayers on iron surface. Appl. Surf. Sci..

[24] Özdemir O.K., Aytaç A., Atilla D., Durmuş M. (2011). Corrosion inhibition of aluminum by novel phthalocyanines in hydrochloric acid solution. J. Mater. Sci..

[25] Sorokin A.B. (2013). Phthalocyanine metal complexes in catalysis. Chem. Rev..

[26] Lokesh K.S., de Keersmaecker M., Elia A., Depla D., Dubruel P., Vandenabeele P., van Vlierberghe S., Adriaens A. (2012). Adsorption of cobalt (II) 5,10,15,20-tetrakis(2-aminophenyl) porphyrin onto copper substrates: Characterization and impedance studies for corrosion inhibition. Corros. Sci..

[27] Kadish K.M., Smith K.M., Guilard R. (2003). The Porphyrin Handbook: Phthalocyanines: Properties and Materials.

[28] Solomon M.M., Umoren S.A., Udousoro I.I., Udoh A.P. (2010). Inhibitive and adsorption behaviour of carboxymethyl cellulose on mild steel corrosion in sulphuric acid solution. Corros. Sci..

[29] Umoren S.A., Ogbobe O., Igwe I.O., Ebenso E.E. (2008). Inhibition of mild steel corrosion in acidic medium using synthetic and naturally occurring polymers and synergistic halides additives. Corros. Sci..

[30] Umoren S.A., Li Y., Wang F.H. (2010). Effect of polyacrylic acid on the corrosion behaviour of aluminium in sulphuric acid solution. J. Solid State Electr..

[31] Qian B., Wang J., Zheng M., Hou B. (2013). Synergistic effect of polyaspartic acid and iodide ion oncorrosion inhibition of mild steel in H_2_SO_4_. Corros. Sci..

[32] Al Fuhaiman L.A., Mustafa A.A., Mekhamer W.K. (2013). Polyvinyl pyrrolidone as a green corrosion inhibitor for carbon steel in alkaline solutions containing NaCl. Anti Corros. Method Mater. J..

[33] Soltani N., Behpour M., Ghoreishi S.M., Naiemi H. (2010). Corrosion inhibition of mild steel in hydrochloric acid solution by some double Schiff bases. Corros. Sci..

[34] Szauer T., Brandt A. (1981). Adsorption of oleates of various amines on iron in acidic solution. Electrochim. Acta.

[35] Dahmani M., Et-Touhami A., Al-Deyab S.S., Hammouti B., Bouyanzer A. (2010). Corrosion inhibition of C38 steel in 1 M HCl: A comparative study of black pepper extract and its isolated piperine. Inter. J. Electrochem. Sci..

[36] Hoar T.P., Holliday R.D. (1953). The inhibition by quinolines and thioureas of the acid dissolution of mild steel. J. Appl. Chem..

[37] Riggs O.L., Hurd R.M. (1967). Temperature coefficient of corrosion inhibition. Corrosion.

[38] Benali O., Larabi L., Merah S., Harek Y. (2011). Influence of the methylene blue dye (MBD) on the corrosion inhibition of mild steel in 0.5 M sulphuric acid, Part I: Weight loss and electrochemical studies. J. Mater. Environ. Sci..

[39] Saliyan V.R., Adhikari A.V. (2007). Inhibition of corrosion of mild steel in acid media by N′-benzylidene-3-(quinolin-4-ylthio)propanohydrazide. Bull. Mater. Sci..

[40] Murulana L.C., Singh A.K., Shukla S.K., Kabanda M.M., Ebenso E.E. (2012). Experimental and quantum chemical studies of some *bis*(trifluoromethyl-sulfonyl) imide imidazolium-based ionic liquids as corrosion inhibitors for mild steel in hydrochloric acid solution. Ind. Eng. Chem. Res..

[41] Asegbeloyi J.N., Ejikeme P.M., Olasunkanmi L.O., Adekunle A.S., Ebenso E.E. (2015). A novel Schiff base of 3-acetyl-4-hydroxy-6-methyl-(2*H*)pyran-2-one and 2,2′-(ethylenedioxy)diethylamine as potential corrosion inhibitor for mild steel in acidic medium. Materials.

[42] Mashuga M.E., Olasunkanmi L.O., Adekunle A.S., Yesudass S., Kabanda M.M., Ebenso E.E. (2015). Adsorption, thermodynamics and quantum chemical studies of 1-hexyl-3-methylimidazolium based ionic liquids as corrosion inhibitors for mild steel in HCl. Materials.

[43] Abdallah M, Asghar B.H., Zaafarany I., Fouda A.S. (2009). The inhibition of carbon steel corrosion in hydrochloric acid solution using some phenolic compounds. Int. J. Electrochem. Sci..

[44] Foad E.E., Abdel Wahaab S.M., Deyab M. (2005). Ethoxylated fatty acids as inhibitors for the corrosion of zinc in acid media. Mater. Chem. Phys..

[45] Gomma G.K. (1998). Corrosion of low carbon steel in sulphuric acid solution in presence of pyrazole-halide mixture. Mater. Chem. Phys..

[46] Umoren S.A., Ogbobe O., Ebenso E.E. (2006). The adsorption characteristics and synergistic inhibition between polyethylene glycol and halides ions for the corrosion inhibition of mild steel in acidic medium. Bull. Electrochem..

[47] Ebenso E.E. (2003). Synergistic effect of halide ions on the corrosion inhibition of aluminium in H_2_SO_4_ using 2-acetylphenothiazine. Mater. Chem. Phys..

[48] Ebenso E.E. (2003). Effect of halide ions on the corrosion inhibition of mild steel in H_2_SO_4_ using methyl red: Part 1. Bull. Electrochem..

[49] Labari L., Harek Y. (2004). Effect of Iodide Ions on Corrosion Inhibition of Mild Steel in 0.5 M H_2_SO_4_ by Poly(4-Vinylpyridine) Port. Electrochim. Acta.

[50] Labari L., Harek Y., Traisnel M., Mansri A. (2004). Synergistic influence of poly(4-Vinylpyridine) and potassium iodide on inhibition of corrosion of mild steel in 1 M HCl. J. Appl. Electrochem..

[51] Yang W., Parr R.G. (1985). Hardness, softness, and the Fukui function in the electronic theory of metals and catalysis. Proc. Natl. Acad. Sci. USA.

[52] Udhayakala P., Rajendiran T.V., Gunasekaran S. (2012). Quantum chemical studies on the efficiencies of vinyl imidazole derivatives as corrosion inhibitors for mild steel. J. Adv. Sci. Res..

[53] Lukovits I., Bakó I., Shaban A., Kálmán E. (1998). Polynomial model of the inhibition mechanism of thiourea derivatives. Electrochim. Acta.

[54] Abreu-Quijano M., Palomar-Pardave M., Cuan A., Romero-Romo M., Negron-Silva G., Alvarez-Bustamante R., Ramirez-Lopez A., Herrera-Hernandez H. (2011). Quantum chemical study of 2-mercaptoimidazole. 2-mercapto-5-methylbenzimidazole and 2-mercapto-5-nitrobenzimidazole as corrosion inhibitors for steel. Int. J. Electrochem. Sci..

[55] Becke A.D. (1993). Density-functional thermochemistry. III. The role of exact exchange. J. Chem. Phys..

[56] Parr R.G., Yang W. (1984). Theoretical organic chemistry. J. Am. Chem. Soc..

[57] Lee C., Yang W., Parr R.G. (1988). Development of the Colle-Salvetti correlation-energy formula into a functional of the electron density. Phys. Rev. B.

[58] Hehre W.J., Random L., Schleyer P.V.R., Pople J.A. (1986). Ab Initio Molecular Orbital Theory.

[59] Wiberg K.B. (2004). Basis Set Effects on Calculated Geometries: 6-311++G** *vs.* aug-cc-pVDZ. J. Comput. Chem..

[60] Brovarets’ O.O., Zhurakivsky R.O., Hovorun D.M. (2014). Does the tautomeric status of the adenine bases change upon the dissociation of the A*.A_syn_ Topal–Fresco DNA mismatch? A combined QM and QTAIM atomistic insight. Phys. Chem. Chem. Phys..

[61] Samijlenko S.P., Yurenko Y.P., Stepanyugin A.V., Hovorun D.M. (2010). Tautomeric equilibrium of approach. J. Phys. Chem. B.

[62] Lozynski M., Rusinska-Roszak D. (1998). Hydrogen bonding and density functional calculations: The B3LYP approach as the shortest way to MP2 results. J. Phys. Chem. A.

[63] Lukovits I., Shaban A., Kalman E. (2003). Corrosion inhibitors: Quantitative structure activity relationships. Russ. J. Electrochem..

[64] (2015). XLSTAT 2015.1.

