# Supplementary material for: Some Phthalocyanine and Naphthalocyanine Derivatives as Corrosion Inhibitors for Aluminium in Acidic Medium: Experimental, Quantum Chemical Calculations, QSAR Studies and Synergistic Effect of Iodide Ions
Source: Molecules. 2015 Aug 28;20(9):15701–34. doi: 10.3390/molecules200915701 (PMC6332286; doi:10.3390/molecules200915701)
Supplement: Supplementary file 1 [file molecules-20-15701-s001.pdf]

Supplementary Materials

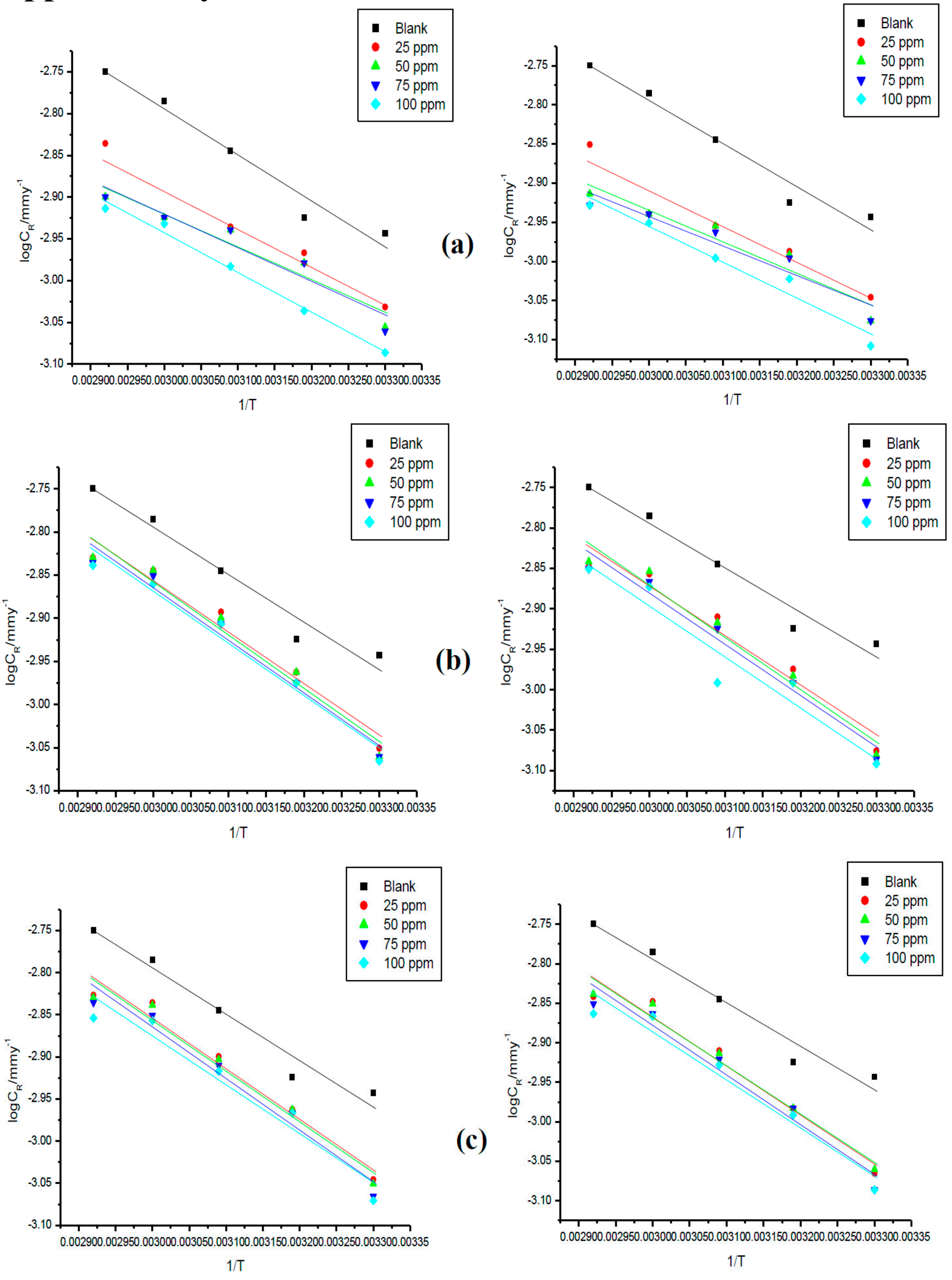

Figure S1. *Cont.*

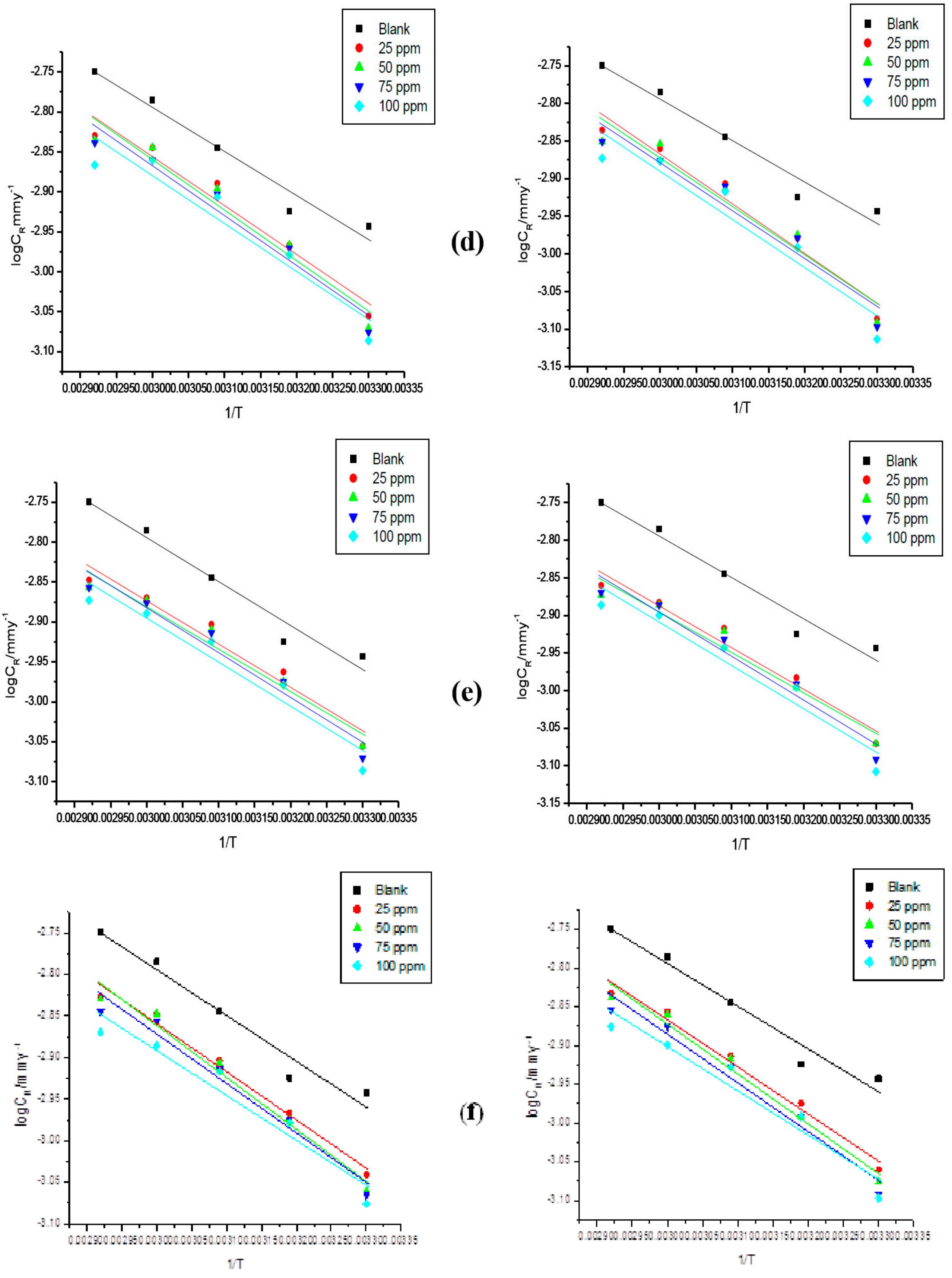

Figure S1. *Cont.*

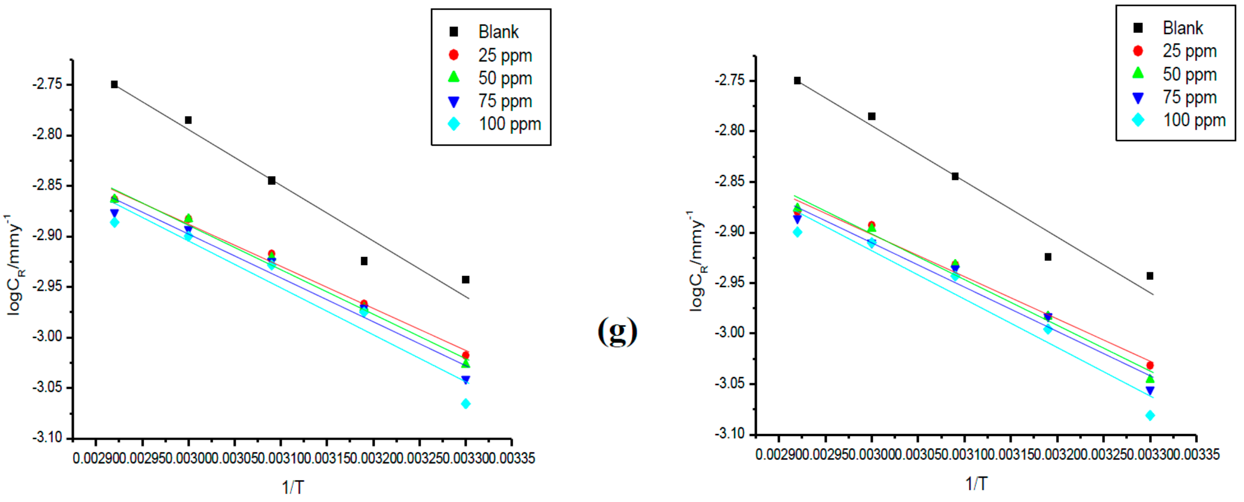

Figure S1. Arrhenius plots (log CR *vs.* 1/T) for Al corrosion in 1 M HCl in the absence and presence of different concentrations of (**a**) Pc1; (**b**) Pc2; (**c**) Pc3; (**d**) Pc4; (**e**) nPc1; (**f**) nPc2; and (**g**) nPc3; without KI (**left hand side**) and with KI (**right hand side**).

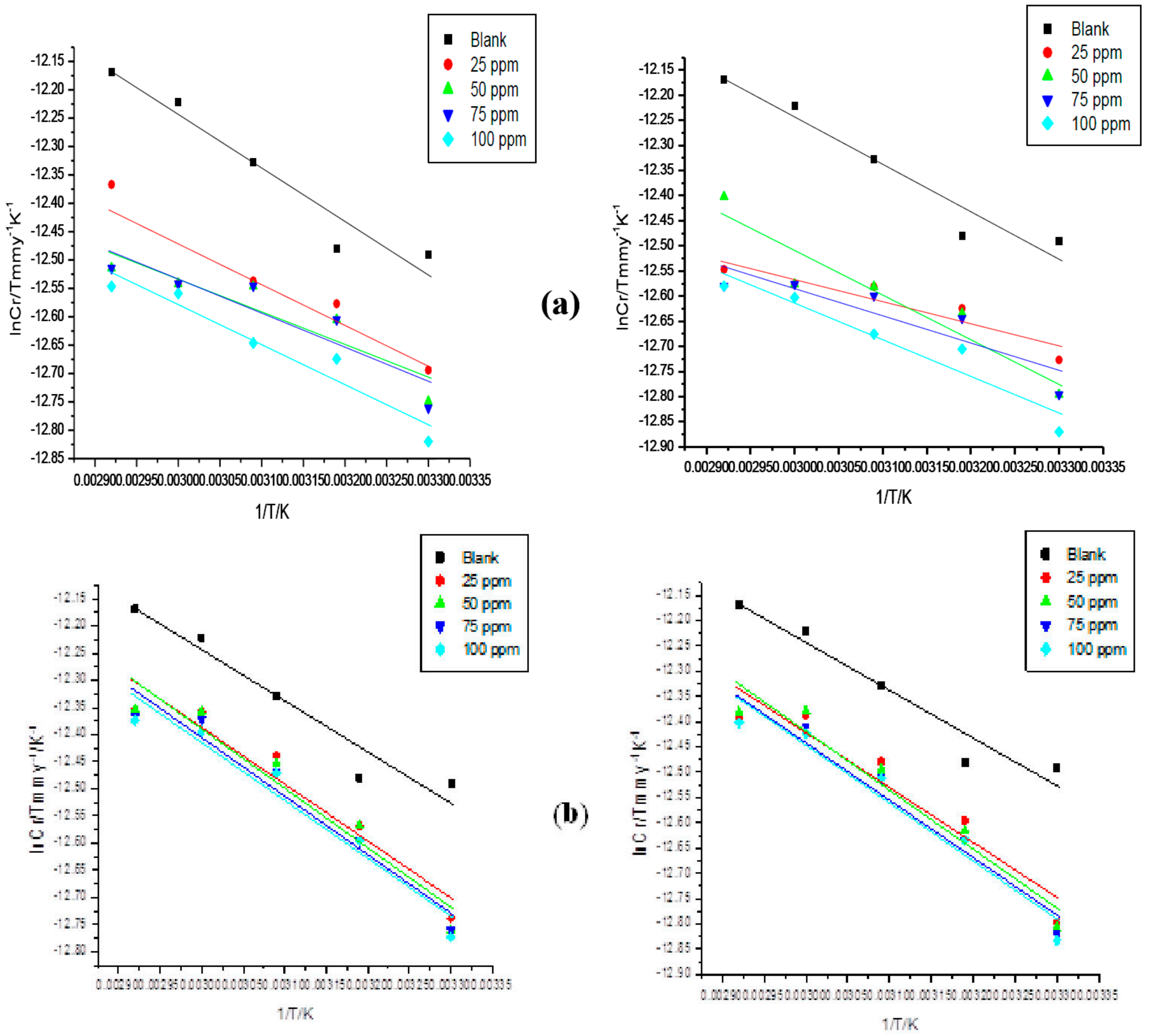

Figure S2. *Cont.*

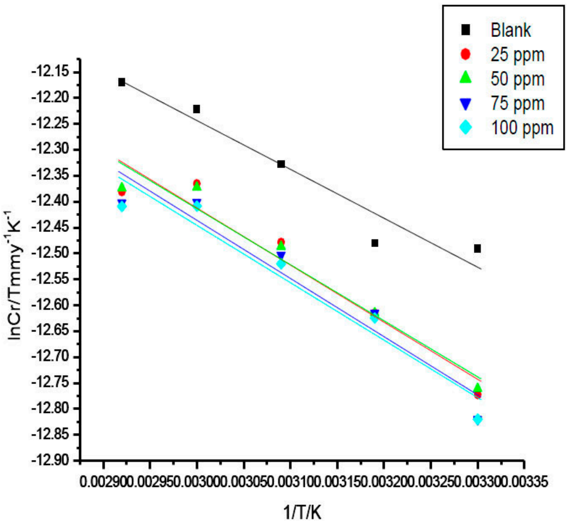

(c)

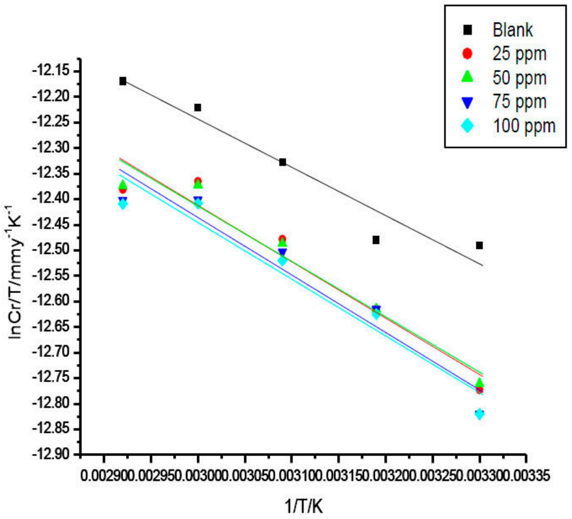

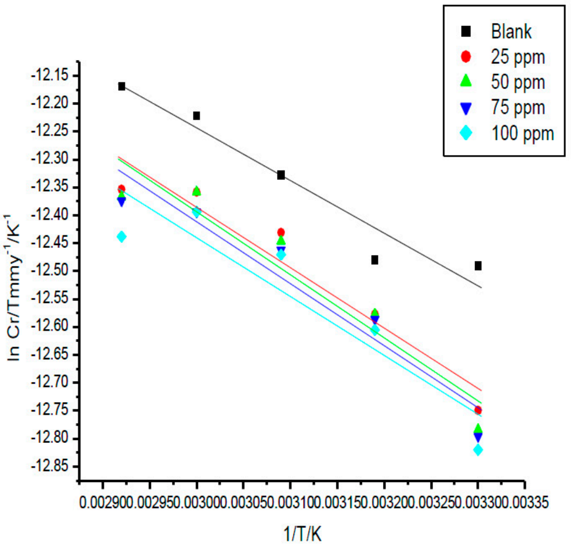

(d)

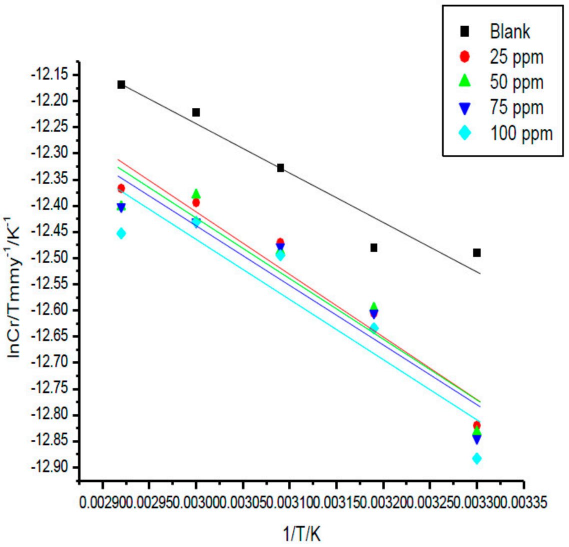

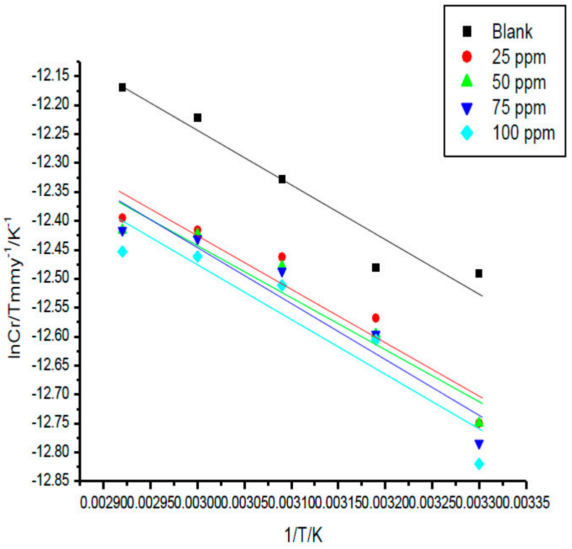

(e)

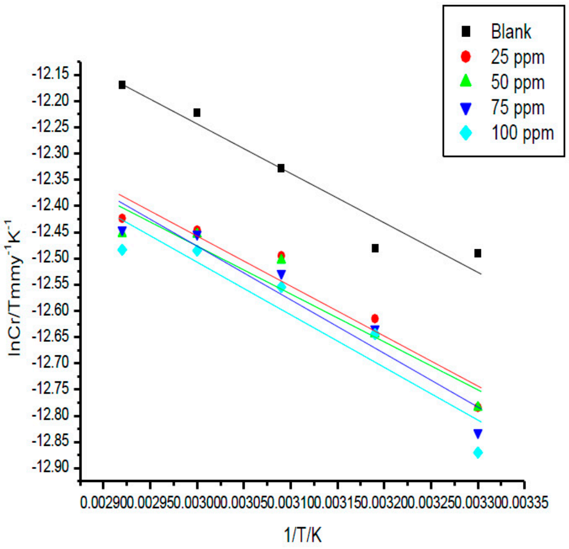

Figure S2. *Cont.*

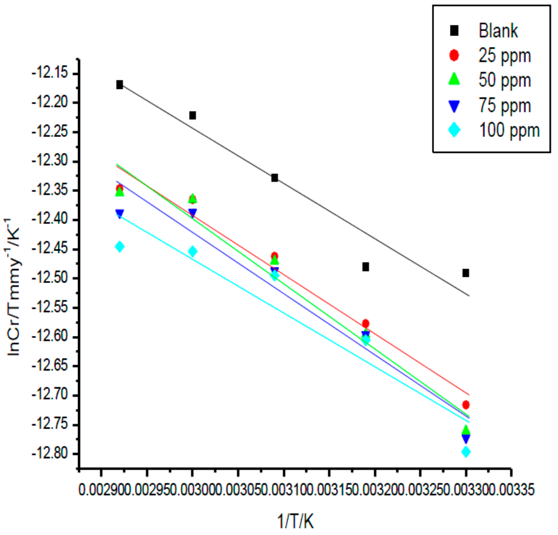

(f)

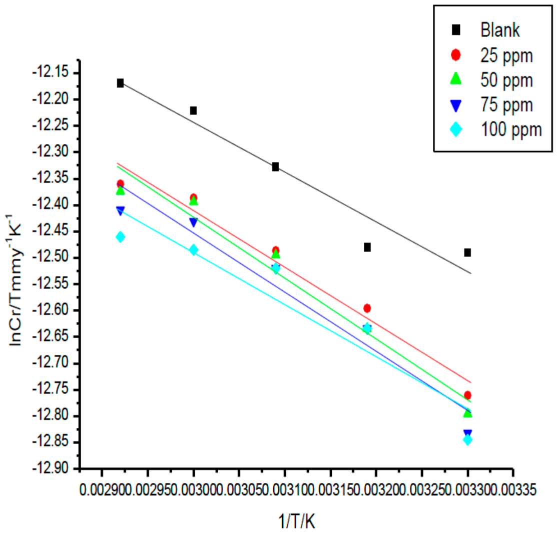

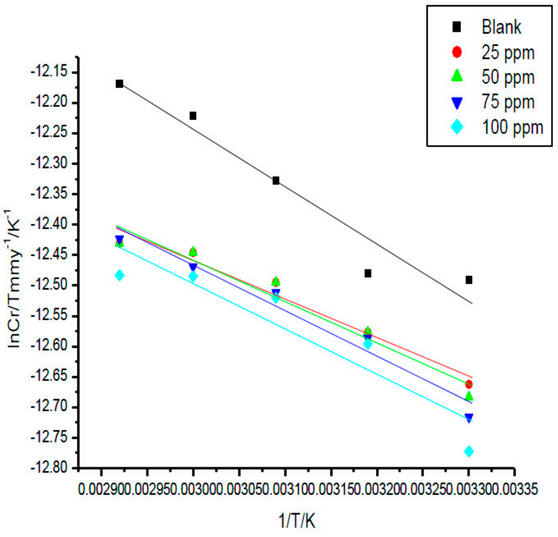

(g)

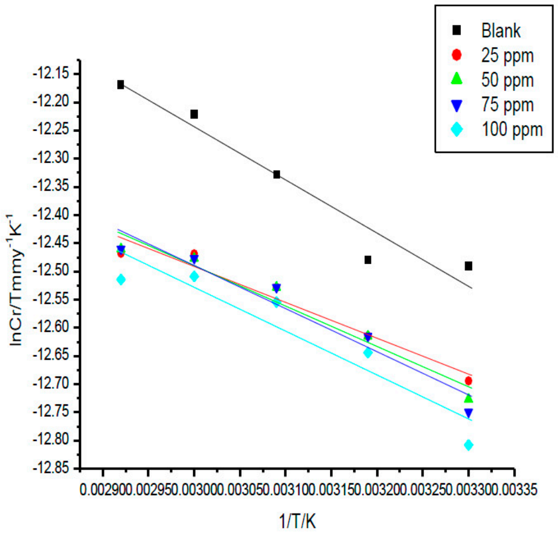

Figure S2. The transition state plots (log C_R/T *vs.* 1/T) for Al corrosion in 1 M HCl in the absence and presence of different concentrations of (**a**) Pc1; (**b**) Pc2; (**c**) Pc3; (**d**) Pc4; (**e**) nPc1; (**f**) nPc2; and (**g**) nPc3; without KI (**left hand side**) and with KI (**right hand side**).

Table S1. Slopes and standard deviations (SD) for the Arrhenius plots (log CR *vs.* 1/T) for Al corrosion in 1 M HCl in the absence and presence of different concentrations of the studied compounds without and with KI.

		Without KI		With KI	
**Inhibitor**	**Conc. (ppm)**	**Slope (× 10^{-2})**	**SD (× 10^{-3})**	**Slope (× 10^{-2})**	**SD (× 10^{-3})**
Blank	-	3.411	7.740	-	-
Pc1	25	2.319	6.240	2.242	6.190
	50	1.942	0.790	1.928	0.938
	75	1.854	0.796	1.794	0.694
	100	2.072	1.970	2.044	1.580
Pc2	25	3.212	2.260	3.175	2.020
	50	3.296	2.220	3.324	2.830
	75	3.210	2.760	3.196	2.520
	100	3.159	2.040	3.201	2.010
Pc3	25	3.247	3.340	3.236	3.410
	50	3.233	3.140	3.188	3.500
	75	3.177	3.460	3.199	2.140
	100	3.015	2.320	3.042	2.550
Pc4	25	3.284	2.340	3.406	1.710
	50	3.352	2.100	3.306	2.280
	75	3.253	1.310	3.220	0.585
	100	3.076	2.720	3.163	1.960
nPc1	25	2.859	0.832	2.822	1.330
	50	2.762	1.560	2.701	2.070
	75	2.881	0.947	2.896	1.260
	100	2.765	0.432	2.780	0.835
nPc2	25	2.859	0.832	2.822	1.330
	50	2.762	1.560	2.701	2.070
	75	2.881	9.468	2.896	1.260
	100	2.765	0.432	2.780	0.835
nPc3	25	2.859	0.832	2.822	1.330
	50	2.763	1.560	2.701	2.070
	75	2.881	0.947	2.896	1.260
	100	2.765	0.432	2.780	0.835

Table S2. * Selected bond lengths (Å), bond angles (degree), dihedral angles, and the symmetry point groups of the studied Pcs and nPcs.

Geometry Parameters	Pc1	Pc2	Pc3	Pc4	nPc1	nPc2	nPc3
N1-H; N9-H	1.013	1.013	1.014	1.014	1.015	1.014	1.014
N3-C4	1.339	1.337	1.336	1.336	1.335	1.337	1.337
N5-C4	1.367	1.366	1.367	1.365	1.364	1.366	1.365
N5-C6	1.361	1.366	1.364	1.365	1.364	1.365	1.365
C6-N7	1.339	1.337	1.337	1.336	1.335	1.337	1.336
N1-C2-N3	126.89	128.24	128.20	128.22	127.80	128.32	128.40
N1-C16-N15	128.20	128.06	128.22	128.26	127.95	128.44	128.40
N7-C8-N9	129.17	128.05	128.23	128.26	127.94	128.44	128.40
N9-C10-N11	126.64	128.24	128.25	128.22	127.82	128.32	128.40
N11-C12-N13	126.57	127.68	127.63	127.71	127.40	127.76	127.80
N13-C14-N15	126.79	127.72	127.71	127.71	127.32	127.79	127.80
C2-N3-C4-N5	−6.81	−0.02	−0.03	−0.22	0.88	−0.30	−0.31
N5-C6-N7-C8	7.09	−0.17	0.15	0.18	0.97	0.02	0.15
N1-C16-N15-C14	10.85	0.23	0.19	0.07	−0.11	0.45	0.23
N9-C10-N11-C12	−8.36	0.06	−0.06	0.15	−1.54	0.47	0.35
** Symmetry point group	C_1	C_i	C_s	D_{2h}	C_1	C_{2h}	D_{2h}

* The selected geometry parameters include only some bond lengths, bond angles and dihedral angles around the common nucleus of the studied Pcs and nPcs. The numbering of atoms displayed in Figure 1 was adopted. ** Schoenflies notations were used for the symmetry point groups.

Table S3. The Mulliken atomic charges on selected * non-hydrogen atoms in Pc1, Pc2, Pc3 and Pc4.

Pc1		Pc2		Pc3		Pc4	
**Atom**	**Charge (*e*)**	**Atom**	**Charge (*e*)**	**Atom**	**Charge (*e*)**	**Atom**	**Charge (*e*)**
N_1	−0.736	N_1	−0.757	N_1	−0.746	N_1	−0.746
N_3	−0.522	N_3	−0.573	N_3	−0.560	N_3	−0.560
N_5	−0.658	N_5	−0.675	N_5	−0.662	N_5	−0.662
N_7	−0.531	N_7	−0.573	N_7	−0.560	N_7	−0.560
O_{1b}	−0.507	O_{1c}	−0.516	C_{1a}	0.064	C_{1a}	0.064
C_{1a}	0.010	C_{1a}	0.061	C_{1b}	−0.182	C_{1b}	−0.182
C_{1b}	0.331	C_{1b}	−0.276	C_{1c}	−0.134	C_{1c}	−0.134
C_{1c}	−0.210	C_{1c}	0.346	C_{5a}	0.082	C_{5a}	0.082
C_{5a}	0.021	C_{5a}	−0.075	C_{5b}	−0.194	C_{5b}	−0.194
C_{5b}	0.310	C_{5b}	−0.284	C_{5c}	−0.134	C_2	0.511
C_{5c}	−0.204	C_{5c}	0.344	C_2	0.511	C_4	0.456
C_2	0.519	C_2	0.509	C_4	0.456	-	-
C_4	0.444	C_4	−0.454	-	-	-	-
C_6	0.449	C_6	0.455	-	-	-	-

* The Mulliken atomic charges are listed for the non-redundant atoms only. Values for equivalent atoms in each molecule differ only by maximum of ±0.003.

Table S4. The Mulliken atomic charges on selected * non-hydrogen atoms in nPc1, nPc2 and nPc3.

**nPc1**		**nPc2**		**nPc3**	
**Atom**	**Charge (*e*)**	**Atom**	**Charge (*e*)**	**Atom**	**Charge (*e*)**
N_1	−0.754	N_1	−0.754	N_1	−0.754
N_3	−0.544	N_3	−0.564	N_3	−0.563
N_5	−0.660	N_5	−0.666	N_5	−0.665
O_{1b}	−0.548	C_{1a}	0.074	C_{1a}	0.075
C_{1a}	0.031	C_{1b}	−0.277	C_{1b}	−0.274
C_{1b}	0.208	C_{1c}	0.153	C_{1c}	0.138
C_{1c}	0.108	C_{1d}	−0.280	C_{1d}	−0.184
C_{1d}	−0.193	C_{1e}	0.183	C_{1e}	−0.137
C_{1e}	−0.136	C_{1f}	−0.189	C_{5a}	0.095
C_{1j}	0.027	C_{1g}	−0.190	C_{5b}	−0.284
C_{5a}	0.048	C_{1h}	0.141	C_2	0.516
C_{5b}	0.196	C_{1i}	−0.277	C_4	0.456
C_{5c}	0.111	C_{1j}	0.073	C_8	0.517
C_{5g}	−0.196	C_{5a}	0.093	-	-
C_2	0.526	C_{5b}	−0.286	-	-
C_4	0.461	C_{5c}	0.141	-	-
C_8	0.527	C_{5d}	−0.192	-	-
-	-	C_{5e}	−0.190	-	-
-	-	C_2	0.515	-	-
-	-	C_4	0.455	-	-

* The Mulliken atomic charges are listed for the non-redundant atoms only. Values for equivalent atoms in each molecule differ only by a maximum of ±0.003.

Table S5. The condensed Fukui functions, f^+ and f^- for Pc1, Pc2, Pc3 and Pc4.

**Pc1**			**Pc2**			**Pc3**			**Pc4**		
**atom**	f^+	f^-	**atom**	f^+	f^-	**atom**	f^+	f^-	**atom**	f^+	f^-
N_1	−0.010	0.005	N_1	−0.012	0.006	N_1	−0.014	0.005	N_1	−0.013	0.005
C_{1a}	0.005	0.001	C_{1a}	−0.006	0.001	C_{1a}	0.002	0.005	C_{1a}	−0.001	0
C_{1b}	−0.016	−0.017	C_{1b}	−0.009	−0.017	C_{1b}	−0.016	−0.020	C_{1b}	−0.01	−0.015
O_{1b}	0.003	−0.006	C_{1c}	−0.012	−0.015	C_{1c}	0.000	0.000	C_{1c}	−0.007	−0.009
C_{1c}	−0.007	−0.013	O_{1c}	−0.012	−0.014	C_{1d}	−0.005	−0.005	C_{1d}	−0.006	−0.009
C_{1d}	−0.011	−0.013	C_{1d}	−0.015	−0.015	C_{1e}	−0.006	−0.008	C_{1e}	−0.375	0.35
C_{1e}	−0.012	−0.018	O_{1d}	−0.01	−0.015	C_{1f}	−0.006	−0.008	C_{1f}	0.128	−0.129
O_{1e}	0.003	−0.007	C_{1e}	−0.009	−0.017	C_{1g}	−0.005	−0.005	C_2	−0.023	−0.04
C_{1f}	−0.008	0.001	C_{1f}	0.000	0.001	C_{1h}	0.000	0.000	N_3	−0.018	−0.006
C_2	−0.033	−0.030	C_2	−0.018	−0.036	C_{1i}	−0.016	−0.02	C_4	−0.042	−0.039
N_3	−0.007	−0.004	N_3	−0.077	0.054	C_{1j}	0.002	0.005	N_5	0.007	0.005
C_4	−0.041	−0.030	C_4	0.869	−0.944	C_2	−0.020	−0.033	C_{5a}	−0.003	0.002
N_5	0.004	0.004	N_5	0.008	0.006	N_3	−0.011	−0.002	C_{5b}	−0.012	−0.016
C_{5a}	0.000	0.001	C_{5a}	−0.003	0.002	C_4	−0.037	−0.032	C_{5c}	−0.01	−0.009
C_{5b}	−0.017	−0.018	C_{5b}	−0.012	−0.017	N_5	0.011	0.034	C_{5d}	−0.01	−0.009
O_{5b}	0.000	−0.006	C_{5c}	−0.020	−0.009	C_{5a}	0.001	0.007	C_{5e}	−0.012	−0.015
C_{5c}	−0.013	−0.012	O_{5c}	−0.011	−0.015	C_{5b}	−0.016	−0.022	C_{5f}	−0.003	0.002
C_{5d}	−0.015	−0.013	C_{5d}	−0.016	−0.014	C_{5c}	−0.002	0.001	C_6	−0.042	−0.039
C_{5e}	−0.015	−0.018	O_{5d}	−0.013	−0.014	C_{5d}	−0.004	−0.006	N_7	−0.017	−0.007
O_{5e}	0.000	−0.008	C_{5e}	−0.014	−0.549	C_{5e}	−0.007	−0.008	C_8	−0.023	−0.04
C_{5f}	−0.008	0.001	C_{5f}	0.147	−0.148	C_{5f}	−0.008	−0.008	N_9	−0.013	0.005
C_6	−0.029	−0.030	C_6	−0.041	−0.035	C_{5g}	−0.003	−0.007	C_{9a}	−0.002	0.002
N_7	−0.068	−0.006	N_7	−0.015	−0.005	C_{5h}	−0.002	0.001	C_{9b}	−0.011	−0.014
C_8	−0.013	−0.030	C_8	−0.022	−0.036	C_{5i}	−0.017	−0.021	C_{9c}	−0.006	−0.009
N_9	−0.009	0.005	N_9	−0.012	0.006	C_{5j}	0.004	0.003	C_{9d}	−0.007	−0.015
C_{9a}	−0.006	0.000	C_{9a}	0.000	0.001	C_6	−0.037	−0.031	C_{9e}	−0.01	0
C_{9b}	−0.013	−0.014	C_{9b}	−0.009	−0.017	N_7	−0.011	−0.002	C_{9f}	−0.001	−0.04
C_{9c}	0.003	−0.003	C_{9c}	−0.015	−0.015	C_8	−0.022	−0.031	C_{10}	−0.023	−0.006
O_{9c}	−0.011	−0.012	O_{9c}	−0.01	−0.015	N_9	−0.014	0.041	N_{11}	−0.018	−0.039
C_{9d}	−0.010	−0.011	C_{9d}	−0.012	−0.015	C_{9a}	0.002	0.005	C_{12}	−0.042	0.005
C_{9e}	−0.010	−0.014	O_{9d}	−0.012	−0.014	C_{9b}	−0.016	−0.020	N_{13}	0.007	0.002
O_{9e}	−0.012	−0.019	C_{9e}	0.018	−0.017	C_{9c}	0.000	0.000	C_{13a}	−0.003	−0.016
C_{9f}	0.001	0.001	C_{9f}	−0.006	0.001	C_{9d}	−0.005	−0.005	C_{13b}	−0.012	−0.009
C_{10}	−0.030	−0.031	C_{10}	−0.018	−0.036	C_{9e}	−0.006	−0.008	C_{13c}	−0.01	−0.009
N_{11}	−0.007	−0.003	N_{11}	−0.017	−0.006	C_{9f}	−0.006	−0.008	C_{13d}	−0.01	−0.015
C_{12}	−0.04	−0.049	C_{12}	−0.039	−0.036	C_{9g}	−0.005	−0.005	C_{13e}	−0.012	0.002
N_{13}	0.003	0.003	N_{13}	0.008	0.006	C_{9h}	0.000	0.000	C_{13f}	−0.003	−0.039
C_{13a}	−0.162	0.163	C_{13a}	−0.003	0.002	C_{9i}	−0.016	−0.02	C_{14}	−0.042	−0.007
C_{13b}	−0.018	−0.019	C_{13b}	−0.012	−0.017	C_{9j}	0.002	0.005	N_{15}	−0.017	−0.04
O_{13b}	0.000	−0.007	C_{13c}	−0.02	−0.014	C_{10}	−0.02	−0.033	C_{16}	−0.023	0.005
C_{13c}	−0.012	−0.014	O_{13c}	−0.011	−0.015	N_{11}	−0.011	−0.002	-	-	-
C_{13d}	−0.015	−0.013	C_{13d}	−0.016	−0.014	C_{12}	−0.037	−0.032	-	-	-
C_{13e}	−0.015	−0.018	O_{13d}	−0.013	−0.014	N_{13}	0.011	0.004	-	-	-
O_{13e}	0.000	-0.008	C_{13e}	-0.014	-0.017	C_{13a}	0.001	0.007	-	-	-
C_{13f}	−0.008	0.001	C_{13f}	−0.003	0.002	C_{13b}	−0.016	−0.022	-	-	-
C_{14}	−0.028	−0.031	C_{14}	−0.041	−0.035	C_{13c}	−0.002	0.001	-	-	-
N_{15}	−0.028	−0.003	N_{15}	−0.015	−0.005	C_{13d}	−0.004	−0.006	-	-	-
C_{16}	−0.014	−0.030	C_{16}	−0.022	−0.036	C_{13e}	−0.007	−0.008	-	-	-

Table S6. The condensed Fukui functions, f^+ and f^- for nPc1, nPc2 and nPc3.

	**nPc1**			**nPc2**			**nPc3**	
**atom**	f^+	f^-	**atom**	f^+	f^-	**atom**	f^+	f^-
N_1	−0.014	0.005	N_1	−0.015	0.006	N_1	−0.014	0.005
C_{1a}	−0.002	0.007	C_{1a}	0.003	0.004	C_{1a}	0.002	0.005
C_{1b}	−0.015	−0.022	C_{1b}	−0.015	−0.019	C_{1b}	−0.016	−0.02
O_{1b}	0.000	−0.004	C_{1c}	0.000	−0.001	C_{1c}	0.000	0.000
C_{1c}	−0.003	−0.002	C_{1d}	−0.007	−0.008	C_{1d}	−0.005	−0.005
C_{1d}	−0.002	−0.007	C_{1e}	−0.004	−0.005	C_{1e}	−0.006	−0.008
C_{1e}	−0.005	−0.007	C_{1f}	−0.009	−0.01	C_{1f}	−0.006	−0.008
C_{1f}	−0.005	−0.007	C_{1g}	−0.004	−0.005	C_{1g}	−0.005	−0.005
C_{1g}	−0.002	−0.007	C_{1h}	0.001	0.001	C_{1h}	0.000	0.000
C_{1h}	−0.004	−0.001	C_{1i}	−0.015	−0.019	C_{1i}	−0.016	−0.020
C_{1i}	−0.018	−0.019	C_{1j}	0.003	0.004	C_{1j}	0.002	0.005
O_{1i}	0.001	−0.005	C_2	−0.02	−0.031	C_2	−0.020	−0.033
C_{1j}	0.006	−0.001	N_3	−0.01	−0.002	N_3	−0.011	−0.002
C_2	−0.018	−0.029	C_4	−0.037	−0.031	C_4	−0.037	−0.032
N_3	−0.011	−0.002	N_5	0.011	0.005	N_5	0.011	0.034
C_4	−0.035	−0.027	C_{5a}	0.003	0.005	C_{5a}	0.001	0.007
N_5	0.1	0.004	C_{5b}	−0.016	−0.02	C_{5b}	−0.016	−0.022
C_{5a}	0.003	0.001	C_{5c}	−0.001	0.001	C_{5c}	−0.002	0.001
C_{5b}	−0.02	−0.021	C_{5d}	−0.003	−0.005	C_{5d}	−0.004	−0.006
O_{5b}	−0.002	−0.005	C_{5e}	−0.011	−0.010	C_{5e}	−0.007	−0.008
C_{5c}	−0.007	−0.001	C_{5f}	−0.006	−0.004	C_{5f}	−0.008	−0.008
C_{5d}	−0.002	−0.005	C_{5g}	−0.006	−0.009	C_{5g}	−0.003	−0.007
C_{5e}	−0.007	−0.006	C_{5h}	−0.003	0.000	C_{5h}	−0.002	0.001
C_{5f}	−0.007	−0.006	C_{5i}	−0.015	−0.020	C_{5i}	−0.017	−0.021
C_{5g}	−0.003	−0.005	C_{5j}	0.002	0.004	C_{5j}	0.004	0.003
C_{5h}	−0.005	−0.004	C_6	−0.036	−0.031	C_6	−0.037	−0.031
C_{5i}	−0.018	−0.022	N_7	−0.010	−0.002	N_7	−0.011	−0.002
O_{5i}	−0.002	−0.005	C_8	−0.020	−0.031	C_8	−0.022	−0.031
C_{5j}	−0.001	0.005	N_9	−0.015	0.006	N_9	−0.014	0.041
C_6	−0.035	−0.028	C_{9a}	0.003	0.004	C_{9a}	0.002	0.005
N_7	−0.011	−0.002	C_{9b}	−0.022	−0.012	C_{9b}	−0.016	−0.020
C_8	−0.02	−0.027	C_{9c}	0.001	0.001	C_{9c}	0.000	0.000
N_9	−0.015	0.005	C_{9d}	−0.004	−0.005	C_{9d}	−0.005	−0.005
C_{9a}	0.006	−0.002	C_{9e}	−0.009	−0.01	C_{9e}	−0.006	−0.008
C_{9b}	−0.019	−0.018	C_{9f}	−0.004	−0.005	C_{9f}	−0.006	−0.008
O_{9b}	−0.001	−0.004	C_{9g}	−0.007	−0.008	C_{9g}	−0.005	−0.005
C_{9c}	−0.001	−0.005	C_{9h}	0	−0.001	C_{9h}	0.000	0.000
C_{9d}	−0.007	−0.001	C_{9i}	−0.015	−0.019	C_{9i}	−0.016	−0.02
C_{9e}	−0.005	−0.007	C_{9j}	0.003	0.004	C_{9j}	0.002	0.005
C_{9f}	−0.006	−0.006	C_{10}	−0.056	−0.031	C_{10}	−0.02	−0.033
C_{9g}	−0.007	−0.002	N_{11}	−0.01	−0.002	N_{11}	−0.011	−0.002
C_{9h}	0.000	−0.006	C_{12}	−0.037	−0.031	C_{12}	−0.037	−0.032
C_{9i}	−0.015	−0.023	N_{13}	0.011	0.005	N_{13}	0.011	0.004
O_{9i}	−0.001	−0.004	C_{13a}	0.003	0.005	C_{13a}	0.001	0.007
C_{9j}	−0.002	0.007	C_{13b}	−0.016	−0.02	C_{13b}	−0.016	−0.022

Table 6. *Cont.*

nPc1			nPc2			nPc3		
**atom**	***f*⁺**	***f*⁻**	**atom**	***f*⁺**	***f*⁻**	**atom**	***f*⁺**	***f*⁻**
C_{10}	−0.019	−0.028	C_{13c}	−0.001	0.001	C_{13c}	−0.002	0.001
N_{11}	−0.011	−0.001	C_{13d}	−0.003	−0.005	C_{13d}	−0.004	−0.006
C_{12}	−0.035	−0.028	C_{13e}	−0.011	−0.01	C_{13e}	−0.007	−0.008
N_{13}	0.010	0.004	C_{13f}	0.358	−0.368	C_{13f}	−0.008	−0.008
C_{13a}	0.002	0.002	C_{13g}	−0.006	−0.009	C_{13g}	−0.003	−0.007
C_{13b}	−0.018	−0.023	C_{13h}	−0.003	0.000	C_{13h}	−0.002	0.001
O_{13b}	−0.003	−0.003	C_{13i}	−0.015	−0.021	C_{13i}	−0.017	−0.021
C_{13c}	−0.007	−0.001	C_{13j}	−0.002	0.009	C_{13j}	0.004	0.003
C_{13d}	−0.003	−0.005	C_{14}	−0.036	−0.031	C_{14}	−0.037	−0.031
C_{13e}	−0.006	−0.007	N_{15}	−0.01	−0.002	N_{15}	−0.011	−0.002
C_{13f}	−0.007	−0.006	C_{16}	−0.02	−0.031	C_{16}	−0.022	−0.031
C_{13g}	−0.003	−0.005						
C_{13h}	−0.004	−0.004						
C_{13i}	−0.02	−0.021						
O_{13i}	−0.001	−0.005						
C_{13j}	0.000	0.003						
C_{14}	−0.035	−0.027						
N_{15}	−0.01	−0.003						
C_{16}	−0.02	−0.027						
